# Supplementary material for: Effects of Origanum vulgare essential oil and its two main components, carvacrol and thymol, on the plant pathogen Botrytis cinerea
Source: PeerJ. 2020 Aug 14;8:e9626. doi: 10.7717/peerj.9626 (PMC7430266; doi:10.7717/peerj.9626)
Supplement: Supplemental Information 1 [file peerj-08-9626-s001.docx]

Table 1 Inhibitory activity of 17 plant essential oils on the mycelial growth of *B*. *cinerea*

| Essential oil | Concentration  （mg/mL） | 2 d | | 4 d | | 6 d | |
| --- | --- | --- | --- | --- | --- | --- | --- |
|  |  | Colony diameter (cm) | Inhibition rate (%) | Colony diameter (cm) | Inhibition  rate (%) | Colony diameter (cm) | Inhibition rate (%) |
| *Cinnamomum cassia* oil | 2 | 0.50±0.00 | 100.00±0.00 | 0.50±0.00 | 100.00±0.00 | 0.50±0.00 | 100.00±0.00 |
|  | 0.5 | 0.50±0.00 | 100.00±0.00 | 0.50±0.00 | 100.00±0.00 | 0.50±0.00 | 100.00±0.00 |
| *Perilla frutescens* oil | 2 | 2.75±0.13 | 7.53±5.44 | 5.75±0.09 | 5.12±1.57 | 8.42±0.08 | -3.71±1.00 |
|  | 0.5 | 2.53±0.03 | 16.44±1.19 | 5.67±0.25 | 6.63±4.55 | 7.75±0.18 | 5.02±2.36 |
| *Saussurea costus* oil | 2 | 1.63±0.13 | 53.42±5.17 | 2.88±0.13 | 56.93±2.27 | 4.53±0.10 | 47.16±1.36 |
|  | 0.5 | 2.32±0.16 | 25.34±6.61 | 4.42±0.03 | 29.22±0.52 | 5.90±0.10 | 29.26±1.31 |
| *Mentha spicata* oil | 2 | 0.50±0.00 | 100.00±0.00 | 0.50±0.00 | 100.00±0.00 | 0.92±0.03 | 94.54±0.38 |
|  | 0.5 | 0.50±0.00 | 100.00±0.00 | 0.50±0.00 | 100.00±0.00 | 1.13±0.23 | 91.70±2.95 |
| *Litsea cubeba* oil | 2 | 0.50±0.00 | 100.00±0.00 | 0.50±0.00 | 100.00±0.00 | 0.50±0.00 | 100.00±0.00 |
|  | 0.5 | 0.50±0.00 | 100.00±0.00 | 0.50±0.00 | 100.00±0.00 | 0.50±0.00 | 100.00±0.00 |
| *Asarum sieboldii* oil | 2 | 0.50±0.00 | 100.00±0.00 | 0.83±0.23 | 93.98±4.17 | 1.83±0.03 | 82.53±0.38 |
|  | 0.5 | 1.10±0.04 | 75.34±1.18 | 2.40±0.05 | 65.66±0.90 | 4.17±0.08 | 51.97±1.00 |
| *Illicium verum* oil | 2 | 0.50±0.00 | 100.00±0.00 | 0.50±0.00 | 100.00±0.00 | 0.53±0.03 | 99.56±0.38 |
|  | 0.5 | 0.62±0.10 | 95.21±4.28 | 2.25±0.05 | 66.57±0.90 | 4.95±0.15 | 41.70±1.97 |
| *Foeniculum vulgare* oil | 2 | 0.55±0.00 | 97.95±0.00 | 1.27±0.44 | 86.14±7.89 | 2.75±0.15 | 70.52±1.97 |
|  | 0.5 | 2.02±0.08 | 37.67±3.14 | 4.42±0.08 | 48.49±3.26 | 5.90±0.10 | 29.26±1.31 |
| *Angelica dahurica* oil | 2 | 1.73±0.08 | 49.32±3.14 | 3.35±0.18 | 48.49±3.26 | 4.45±0.40 | 48.25±5.20 |
|  | 0.5 | 2.05±0.09 | 36.30±3.56 | 4.25±0.18 | 32.23±3.26 | 6.07±0.43 | 27.07±5.65 |
| *Curcuma zedoaria* oil | 2 | 1.12±0.08 | 74.66±3.14 | 2.95±0.09 | 55.72±1.57 | 4.70±0.26 | 44.98±3.40 |
|  | 0.5 | 1.40±0.10 | 63.01±4.11 | 3.55±0.18 | 44.88±3.26 | 5.30±0.52 | 5.58±0.13 |
| *Mentha haplocalyx* oil | 2 | 0.50±0.00 | 100.00±0.00 | 0.50±0.00 | 100.00±0.00 | 0.50±0.00 | 100.00±0.00 |
|  | 0.5 | 0.70±0.17 | 91.78±7.12 | 1.23±0.23 | 86.75±4.07 | 3.55±0.10 | 60.04±1.31 |
| *Artemisia argyi*  oil | 2 | 0.82±0.01 | 86.99±0.59 | 2.88±0.23 | 56.93±4.07 | 6.18±0.13 | 25.66±1.64 |
|  | 0.5 | 2.97±0.08 | -1.37±3.14 | 5.82±0.21 | 3.92±3.76 | 7.80±0.10 | 4.37±1.31 |
| *Eucalyptus globulus* oil | 2 | 1.15±0.05 | 73.29±2.05 | 4.00±000 | 36.75±0.00 | 4.68±0.00 | 45.24±0.00 |
|  | 0.5 | 2.98±0.28 | -1.71±11.30 | 5.75±0.00 | 5.12±0.00 | 7.03±0.00 | 14.52±0.00 |
| *Syzygium aromaticum* oil | 2 | 0.50±0.00 | 100.00±0.00 | 0.50±0.00 | 100.00±0.00 | 1.20±0.05 | 90.83±0.66 |
|  | 0.5 | 0.50±0.00 | 100.00±0.00 | 0.50±0.00 | 100.00±0.00 | 1.45±0.09 | 87.55±1.13 |
| *Acorus tatarinowii* oil | 2 | 2.80±0.18 | 5.48±7.41 | 6.03±0.20 | 0.00±3.65 | 8.30±0.18 | -2.18±2.36 |
|  | 0.5 | 3.15±0.05 | -8.90±2.05 | 6.52±0.26 | -8.73±4.64 | 8.55±0.09 | -5.46±1.13 |
| *Origanum vulgare*  oil | 2 | 0.50±0.00 | 100.00±0.00 | 0.50±0.00 | 100.00±0.00 | 0.50±0.00 | 100.00±0.00 |
|  | 0.5 | 0.50±0.00 | 100.00±0.00 | 0.50±0.00 | 100.00±0.00 | 0.50±0.00 | 100.00±0.00 |
| turpentine oil | 2 | 3.00±0.10 | -2.74±4.11 | 5.75±0.00 | 5.12±0.00 | 8.50±0.05 | -4.80±0.66 |
|  | 0.5 | 2.98±0.13 | -2.05±5.17 | 6.65±0.13 | -11.14±2.39 | 8.43±0.21 | -3.93±2.73 |
| Control | - | 2.93±0.10 | - | 6.03±0.23 | - | 8.13±0.10 | - |
