# Supplementary material for: Effects of Origanum vulgare essential oil and its two main components, carvacrol and thymol, on the plant pathogen Botrytis cinerea
Source: PeerJ. 2020 Aug 14;8:e9626. doi: 10.7717/peerj.9626 (PMC7430266; doi:10.7717/peerj.9626)
Supplement: Supplemental Information 2 [file peerj-08-9626-s002.docx]

Table 2 Effects of OVEO, carvacrol and thymol on the spore germination of *B*. *cinerea*

| Concentration  (μg/mL) | Spore germination (%) | | |
| --- | --- | --- | --- |
|  | OVEO | Carvacrol | Thymol |
| 50 | 100.00±0.00 a | 90.51±0.24 b | 87.98±1.98 b |
| 150 | 100.00±0.00 a | 79.53±1.28 c | 79.44±2.18 c |
| 200 | 100.00±0.00 a | 25.54±2.05 d | 13.25±2.98 d |
| 250 | 89.82±0.31 b | 1.67±0.56 e | 0.00±0.00 e |
| 300 | 80.03±1.15 c | 0.00±0.00 e | 0.00±0.00 e |
| CK | 100.00±0.00 a | 100.00±0.00 a | 100.00±0.00 a |
