# Supplementary material for: Effects of Origanum vulgare essential oil and its two main components, carvacrol and thymol, on the plant pathogen Botrytis cinerea
Source: PeerJ. 2020 Aug 14;8:e9626. doi: 10.7717/peerj.9626 (PMC7430266; doi:10.7717/peerj.9626)
Supplement: Supplemental Information 3 [file peerj-08-9626-s003.docx]

Table 3 Effects of carvacrol and thymol against mycelial biomass of *B*. *cinerea*.

| Regents | Concentration  (μg/mL) | Fresh weight  (mg) | Inhibition rate (%) | Dry weight  (mg) | Inhibition rate (%) |
| --- | --- | --- | --- | --- | --- |
| Carvacrol | EC_50_ | 21.9±2.21b | 86.88±1.32a | 8.30±0.96b | 78.53±2.49a |
|  | EC_90_ | 5.83±1.66c | 96.51±0.99b | 4.13±0.96c | 89.31±2.49b |
|  | CK | 167.17±8.43a | - | 38.67±2.15a | - |
| Thymol | EC_50_ | 37.50±1.20b | 78.06±0.41a | 11.32±0.70b | 69.94±1.02a |
|  | EC_90_ | 3.03±0.21c | 98.23±0.05b | 1.52±0.03c | 95.97±0.07b |
|  | CK | 170.90±12.15a | - | 37.64±1.61a | - |
