# Supplementary figures and images for: Effects of Origanum vulgare essential oil and its two main components, carvacrol and thymol, on the plant pathogen Botrytis cinerea
Source: PeerJ. 2020 Aug 14;8:e9626. doi: 10.7717/peerj.9626 (PMC7430266; doi:10.7717/peerj.9626)

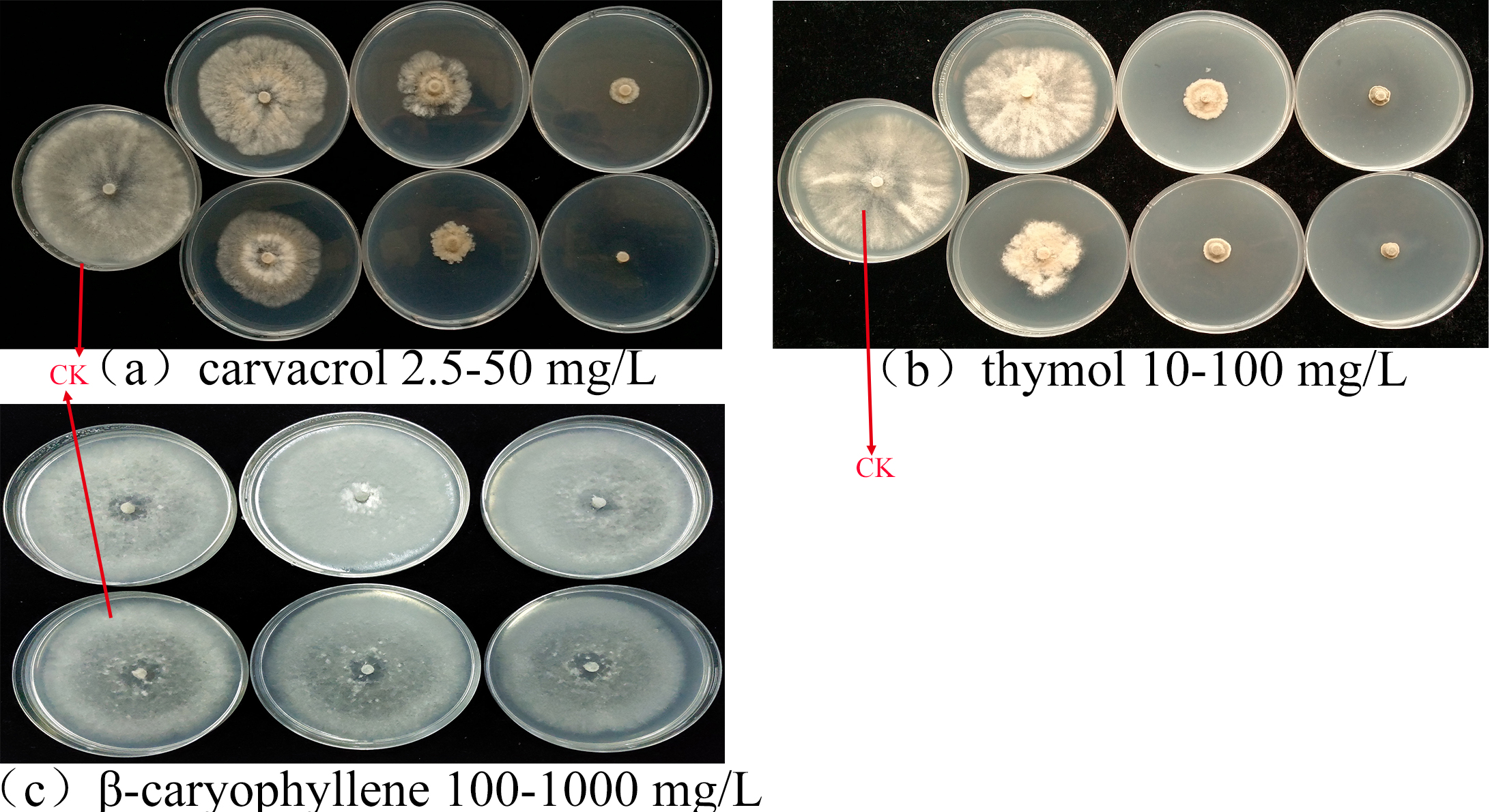

Supplement: Supplemental Information 4 [file peerj-08-9626-s004.jpg]

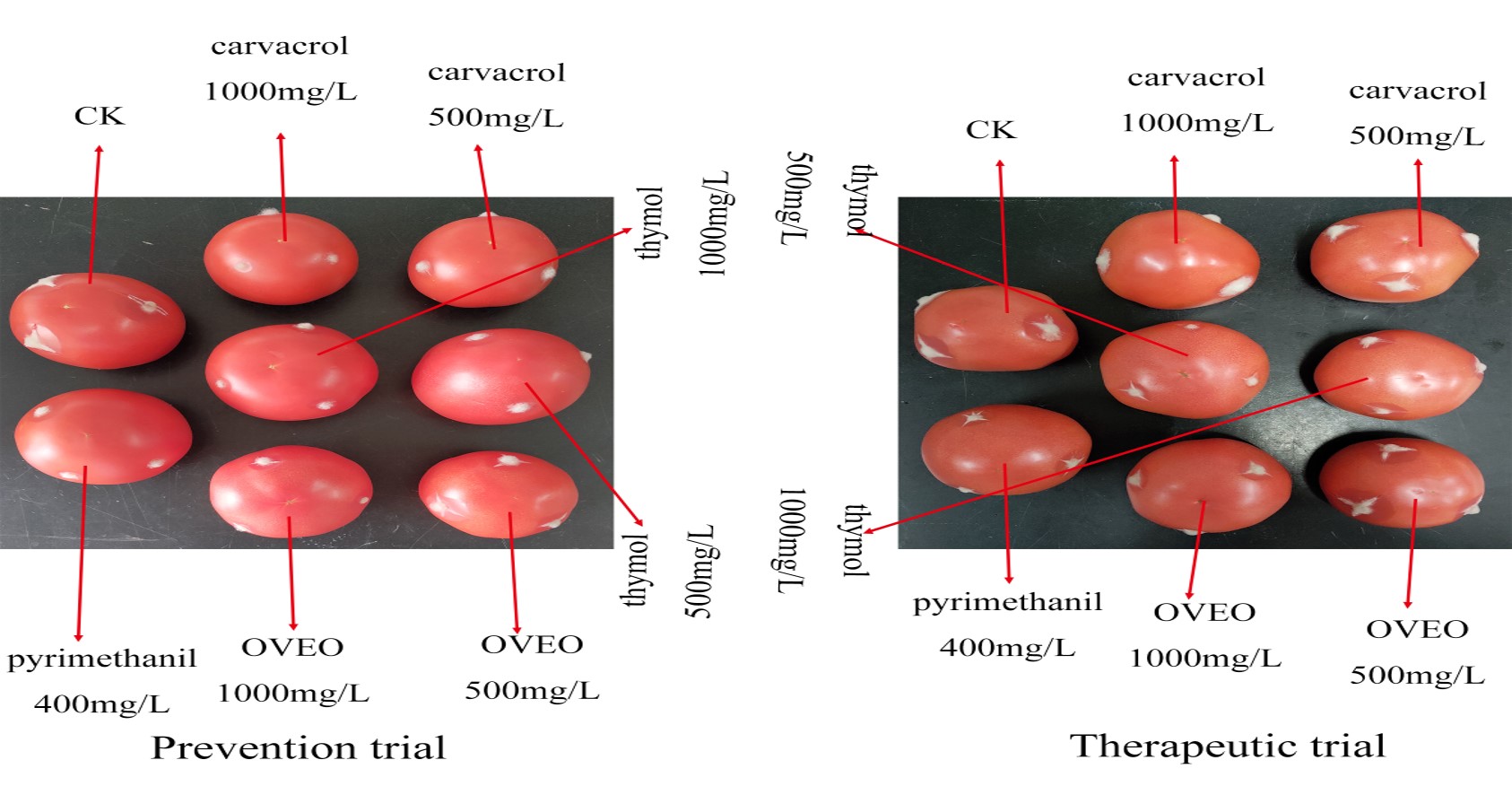

Supplement: Supplemental Information 5 [file peerj-08-9626-s005.jpg]

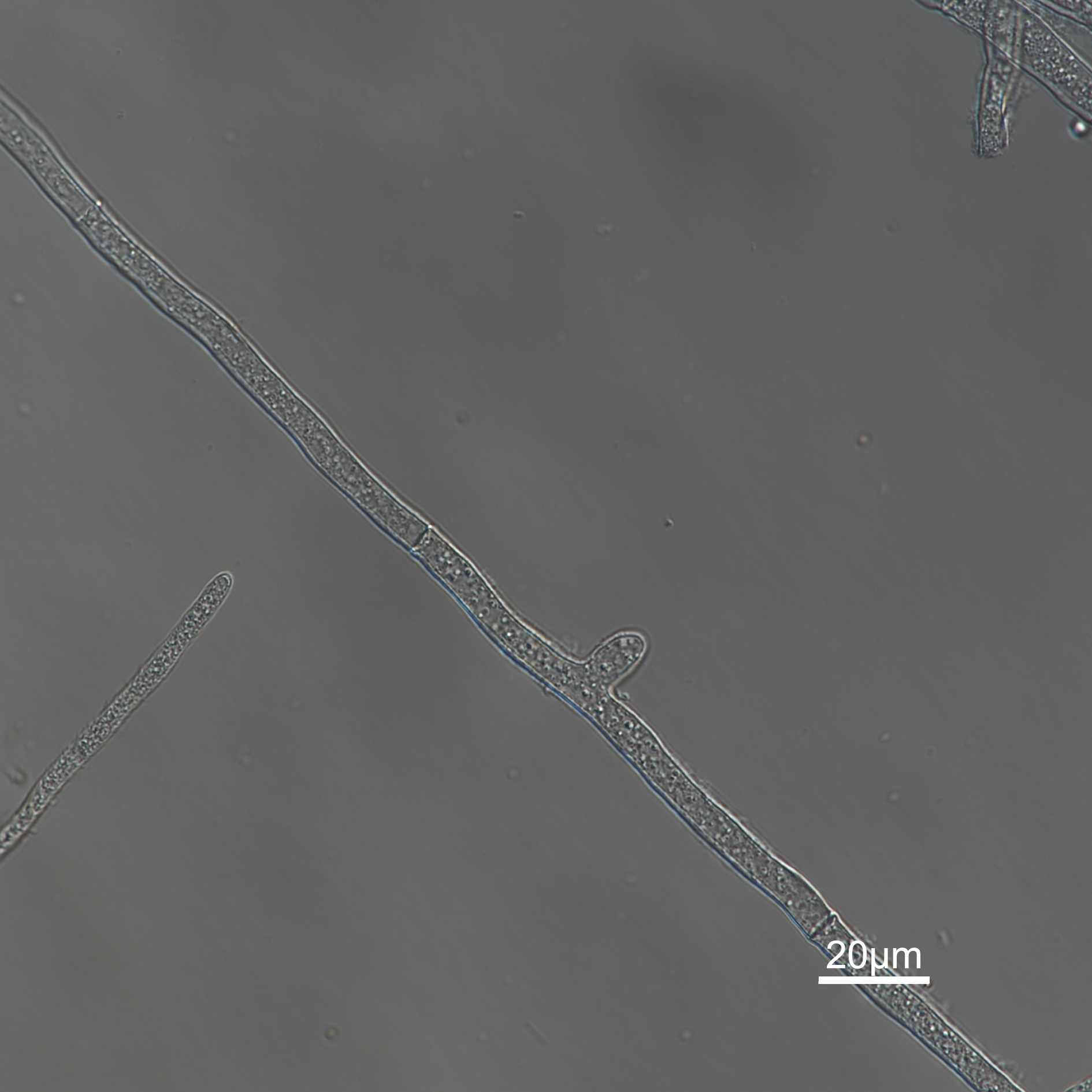

Supplement: Supplemental Information 9 [file peerj-08-9626-s009.zip › fungal membrane integrity/carvacrol bright field.jpg]

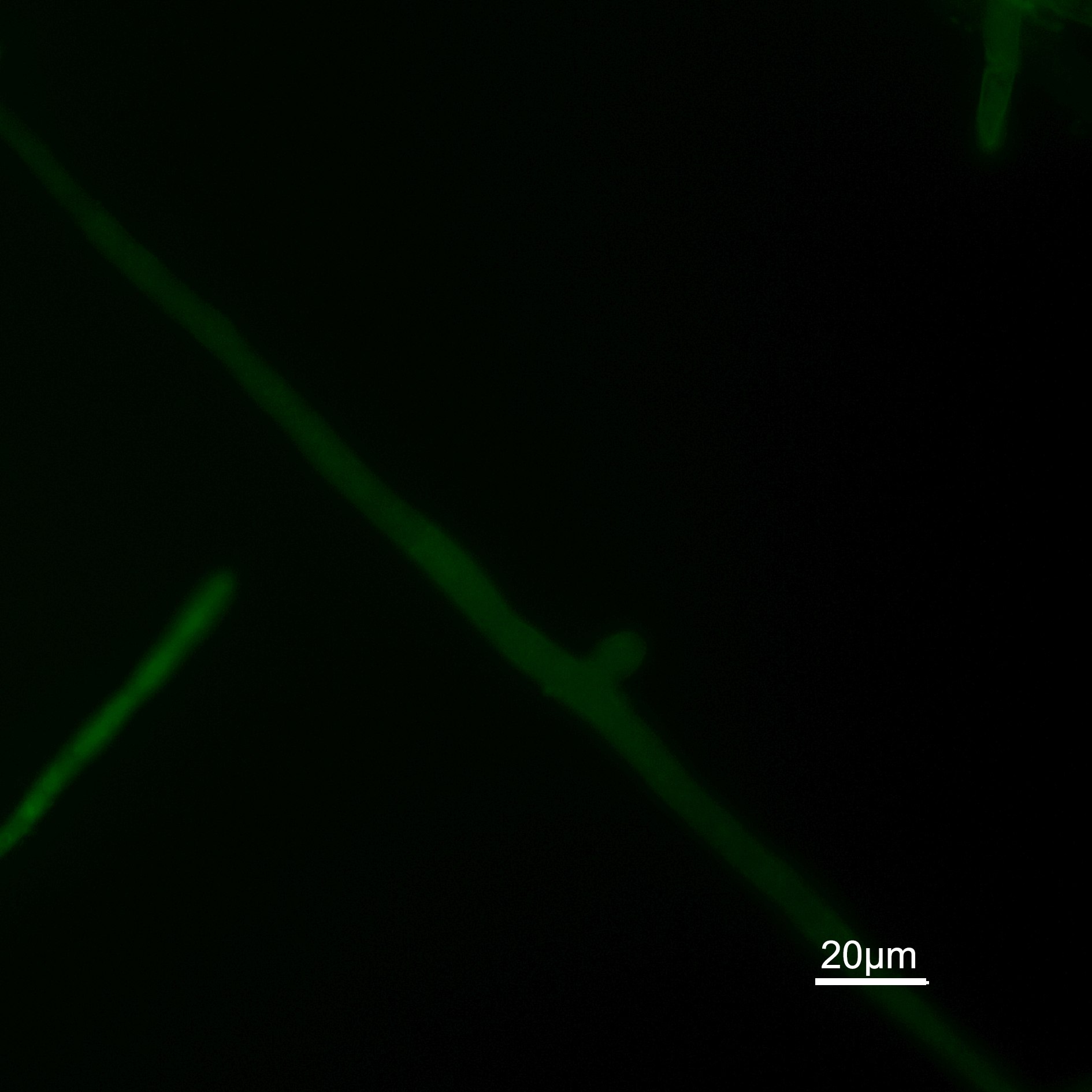

Supplement: Supplemental Information 9 [file peerj-08-9626-s009.zip › fungal membrane integrity/carvacrol with SYTOX Green.jpg]

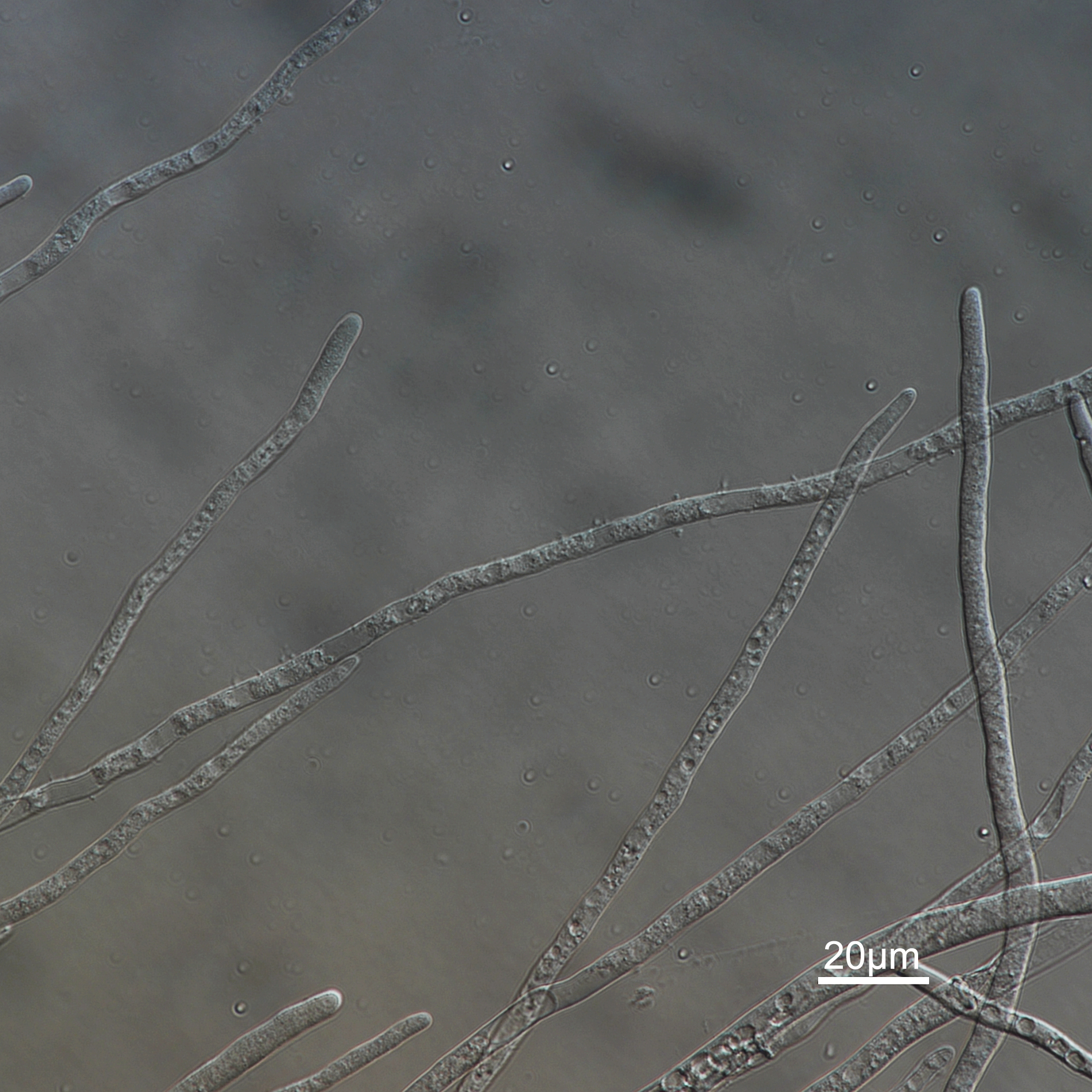

Supplement: Supplemental Information 9 [file peerj-08-9626-s009.zip › fungal membrane integrity/contol bright field.jpg]

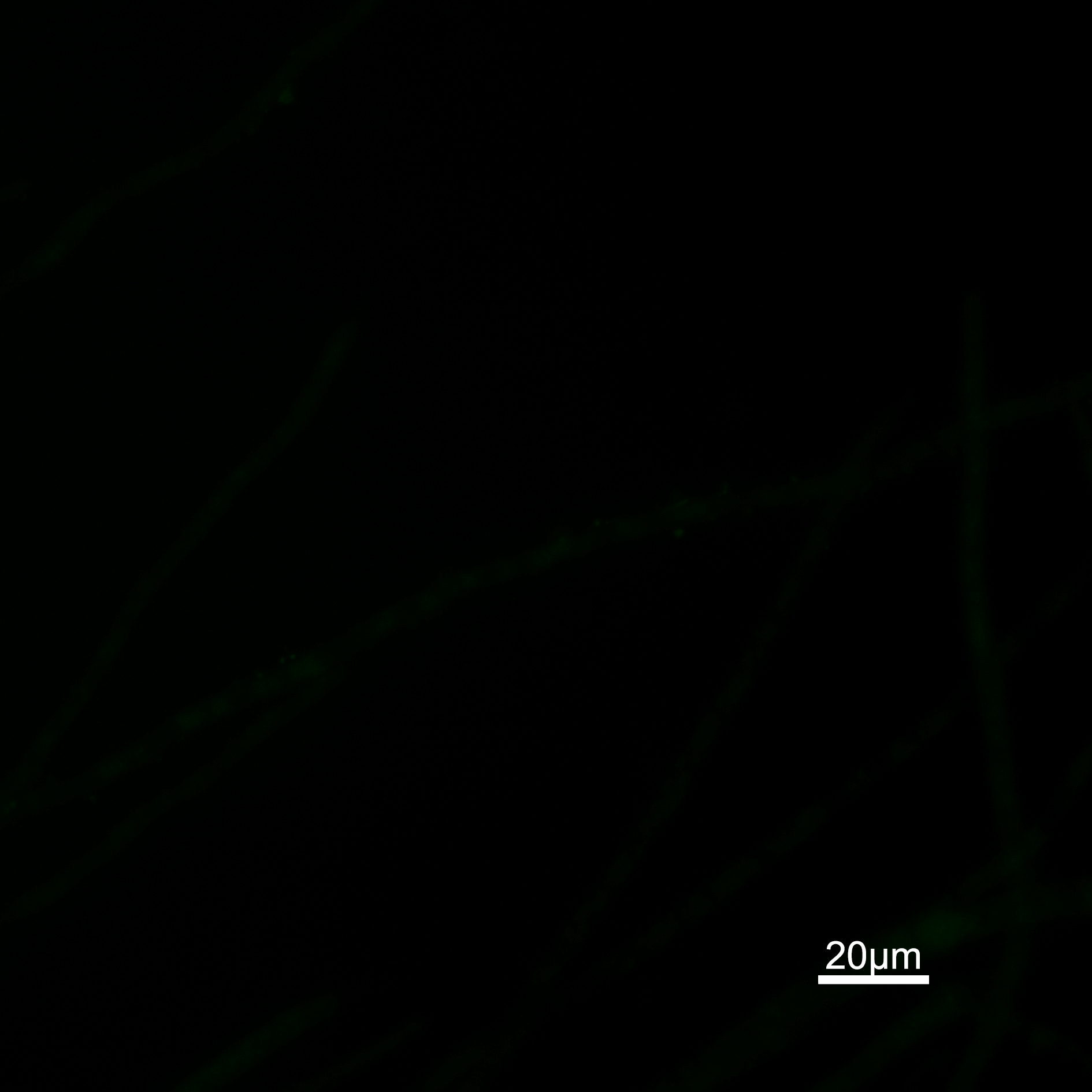

Supplement: Supplemental Information 9 [file peerj-08-9626-s009.zip › fungal membrane integrity/control with SYTOX Green.jpg]

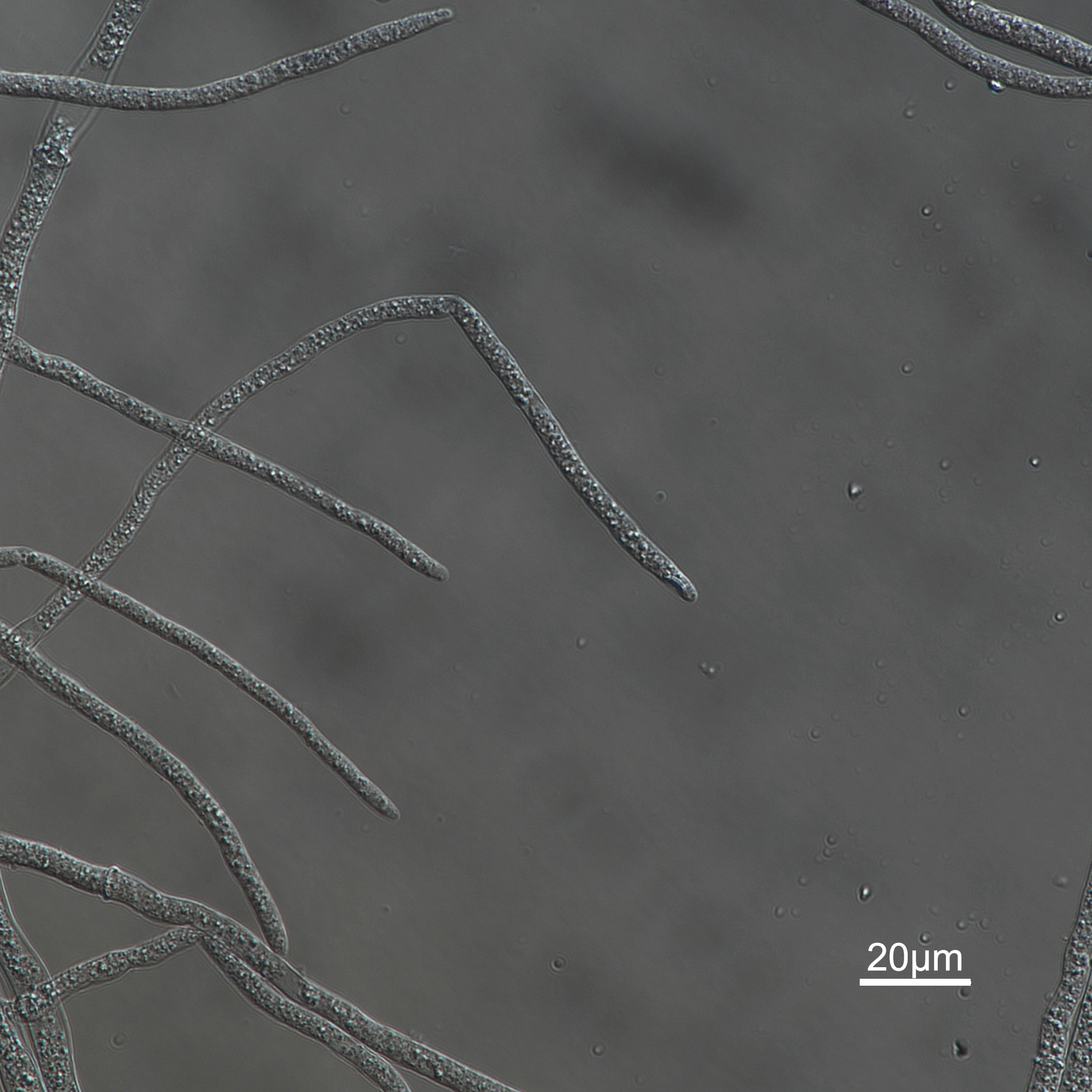

Supplement: Supplemental Information 9 [file peerj-08-9626-s009.zip › fungal membrane integrity/thymol bright field.jpg]

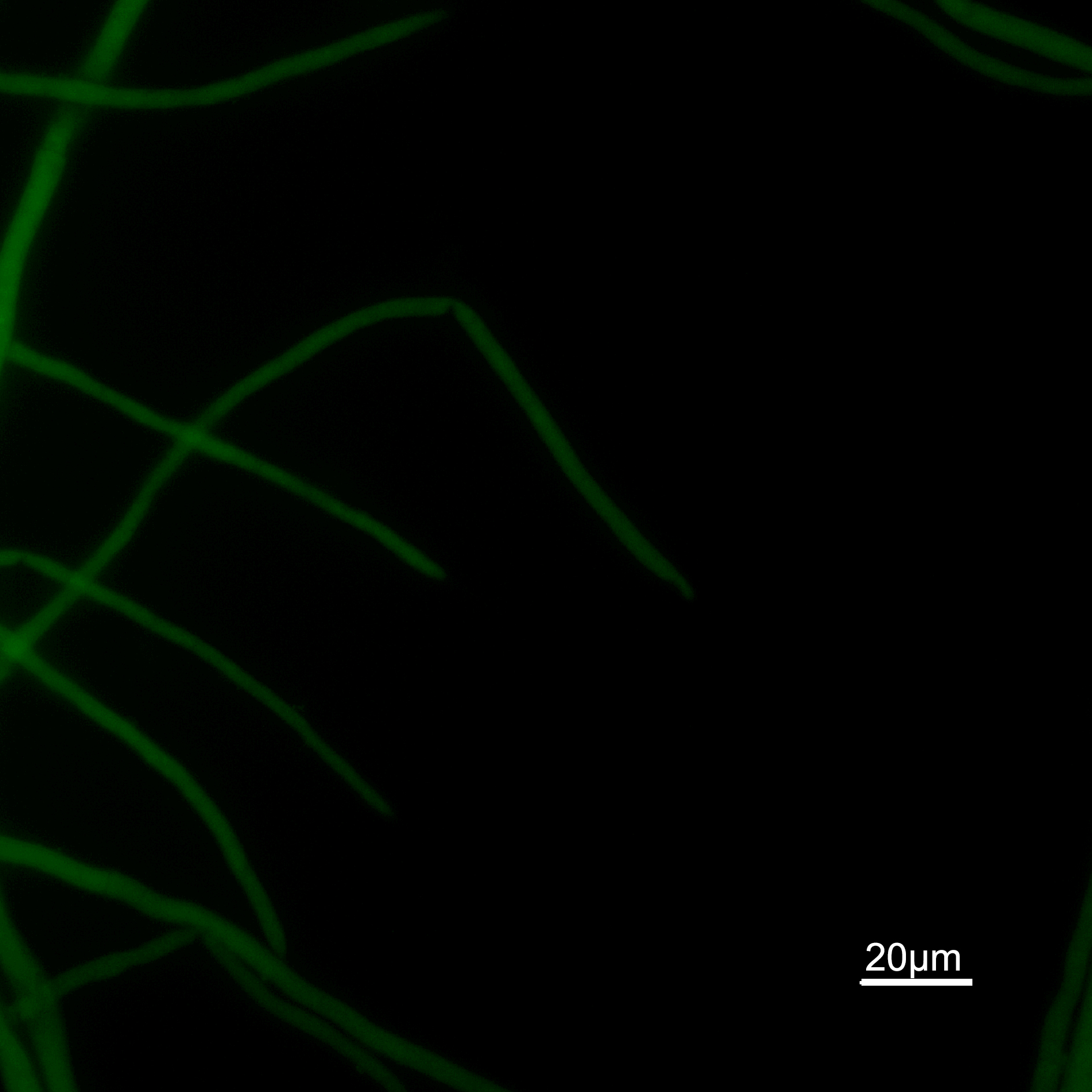

Supplement: Supplemental Information 9 [file peerj-08-9626-s009.zip › fungal membrane integrity/thymol with SYTOX Green.jpg]

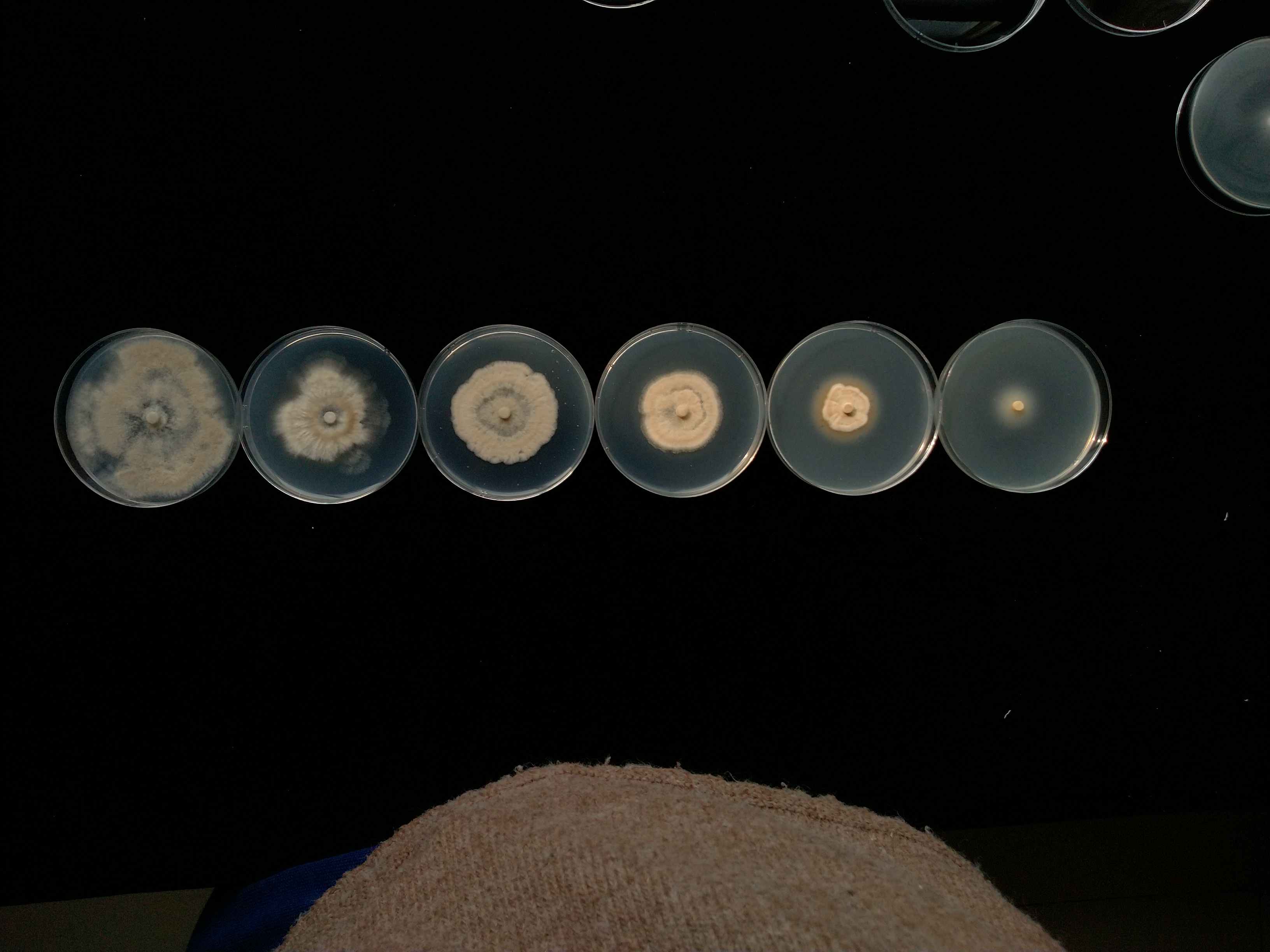

Supplement: Supplemental Information 10 [file peerj-08-9626-s010.zip › EC50valuas/O. vulgare1.jpg]

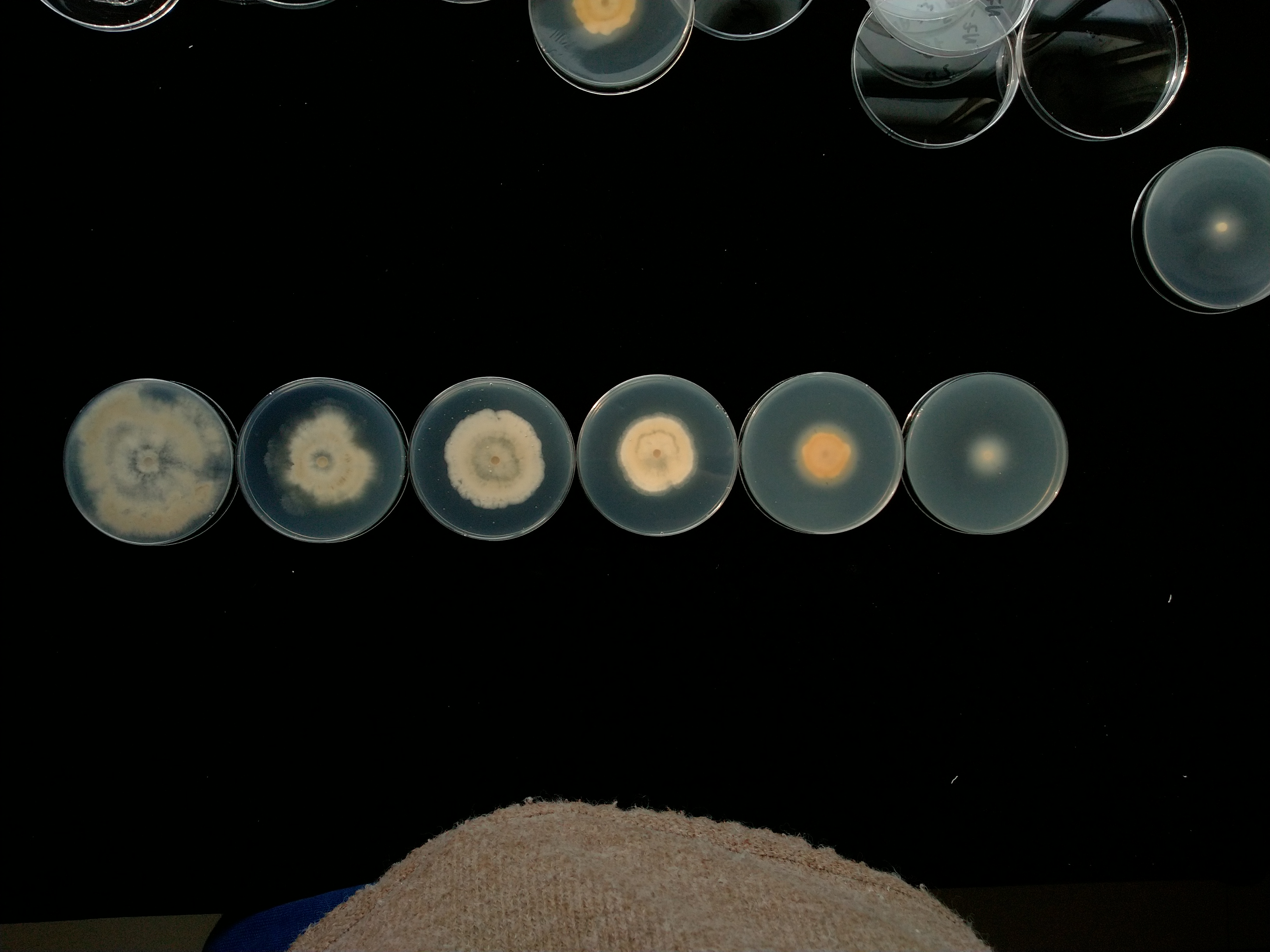

Supplement: Supplemental Information 10 [file peerj-08-9626-s010.zip › EC50valuas/O. vulgare2.jpg]

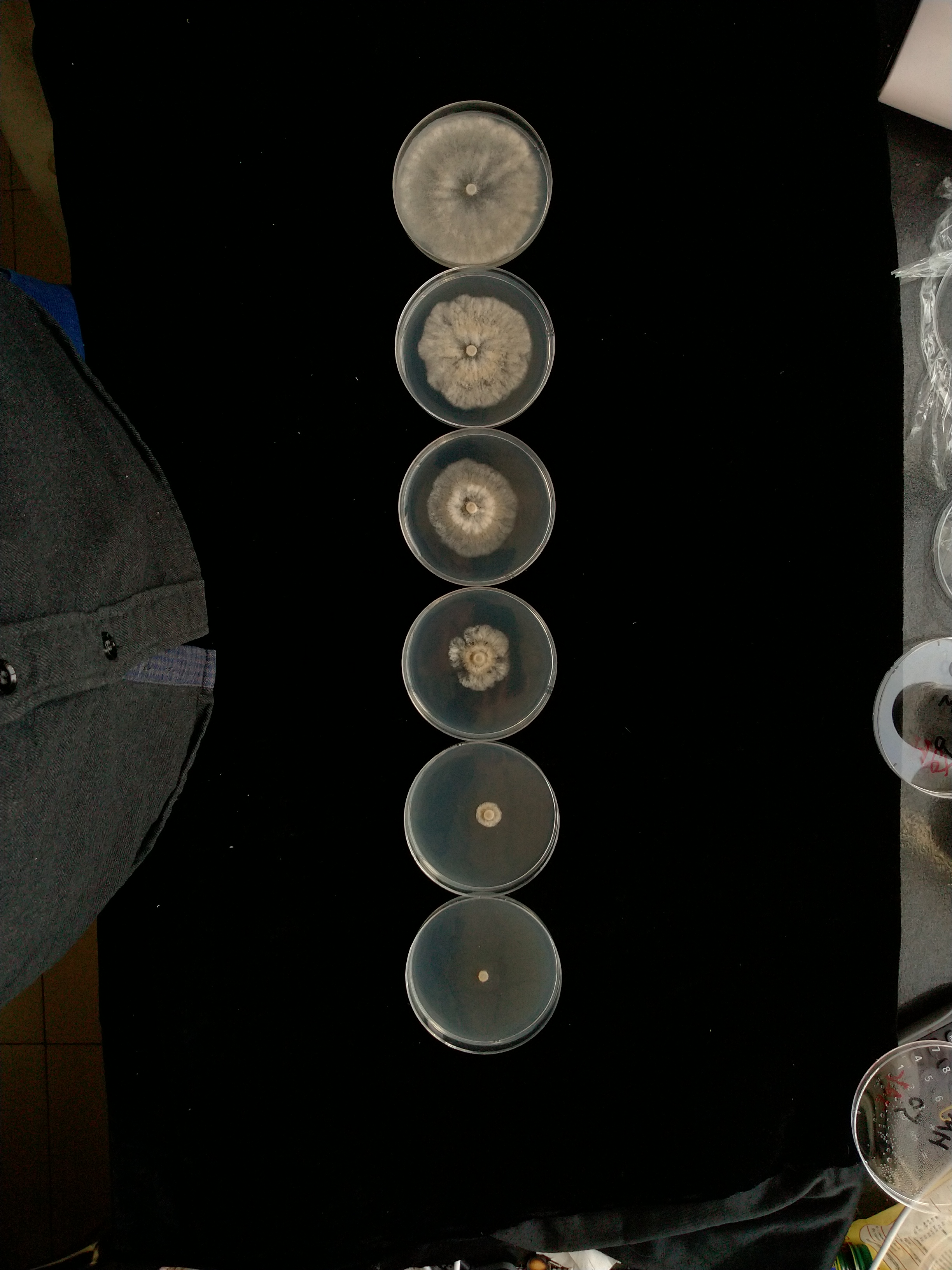

Supplement: Supplemental Information 10 [file peerj-08-9626-s010.zip › EC50valuas/carvacrol1.jpg]

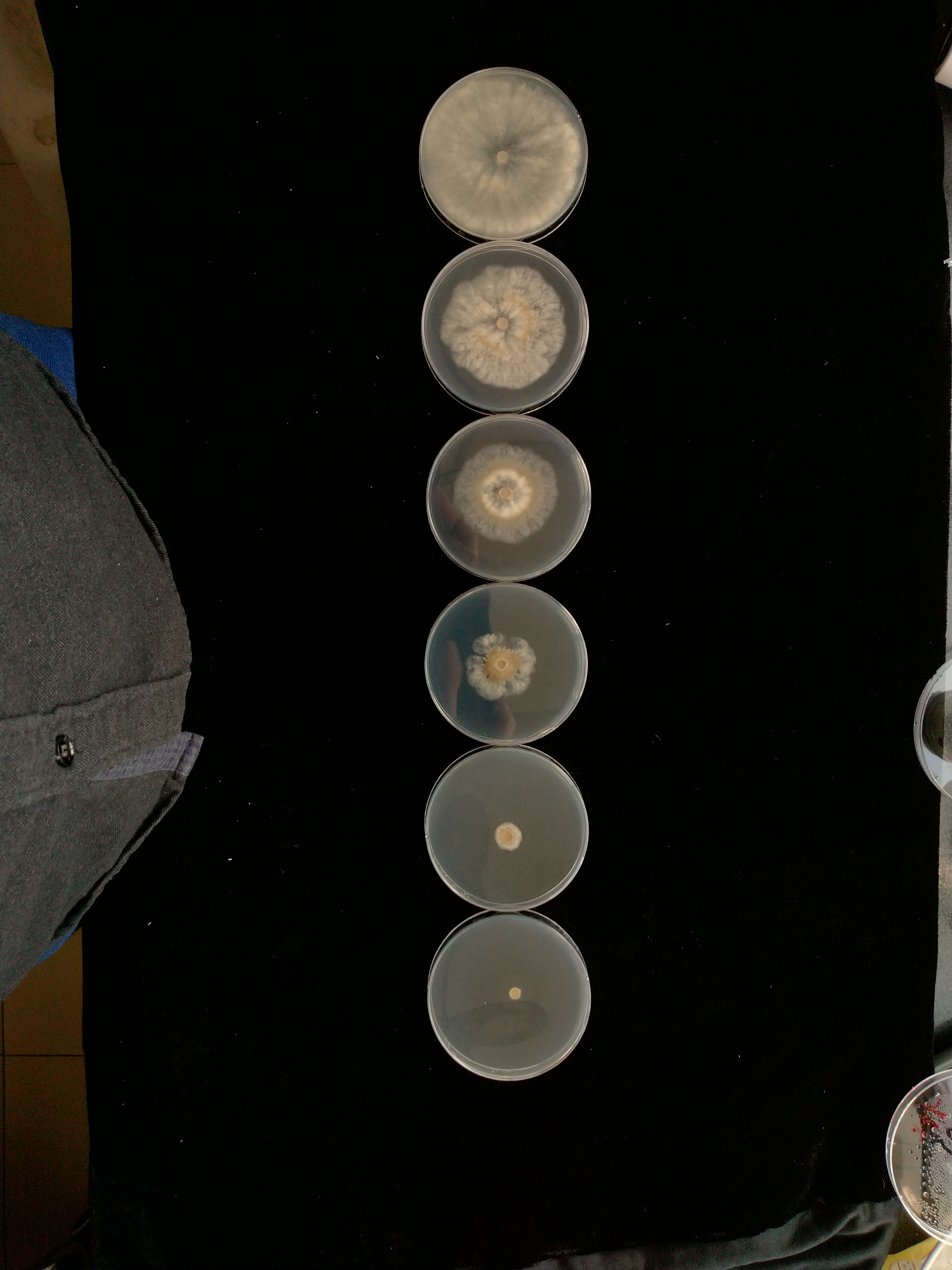

Supplement: Supplemental Information 10 [file peerj-08-9626-s010.zip › EC50valuas/carvacrol2.jpg]

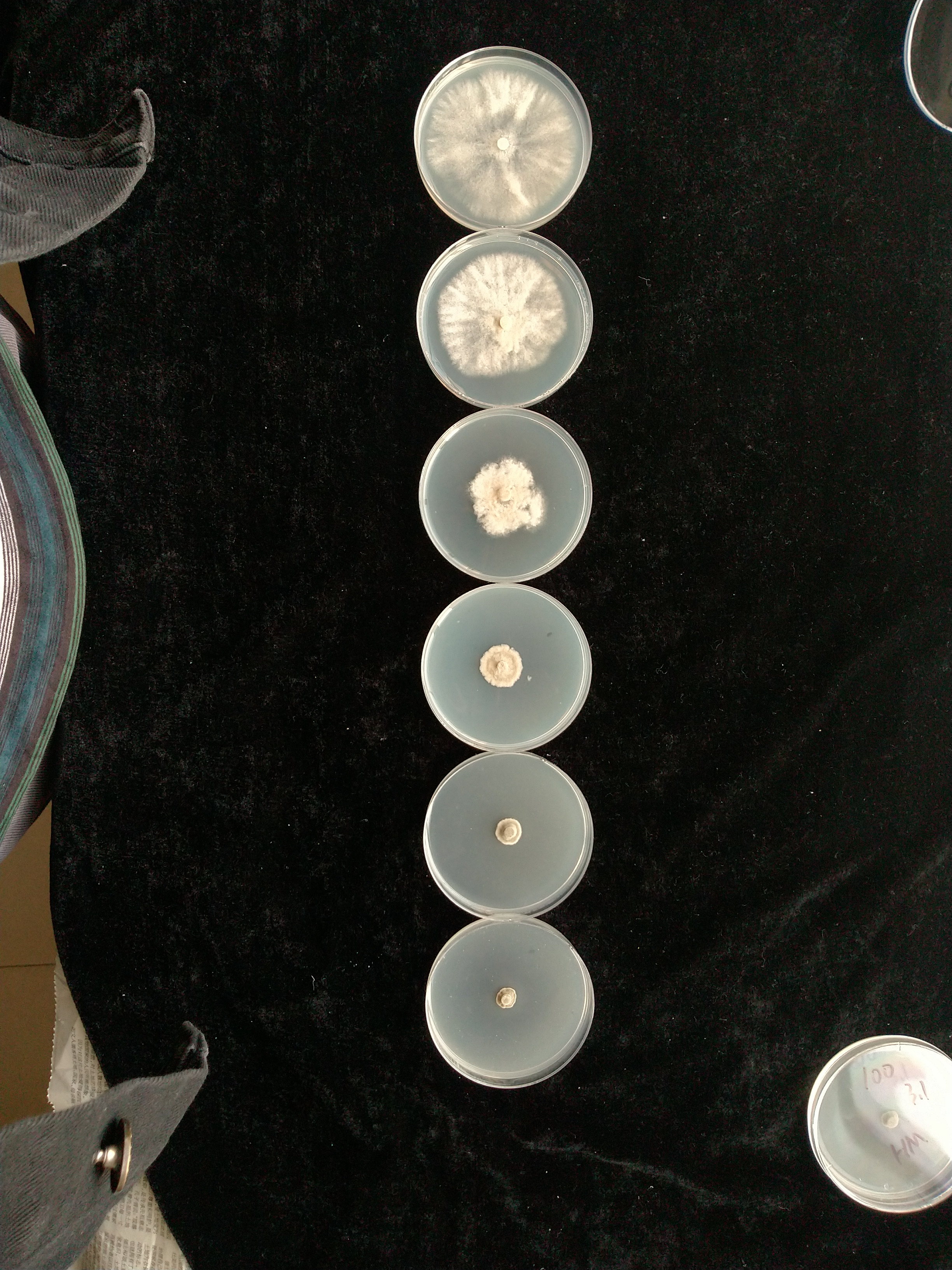

Supplement: Supplemental Information 10 [file peerj-08-9626-s010.zip › EC50valuas/thymol1.jpg]

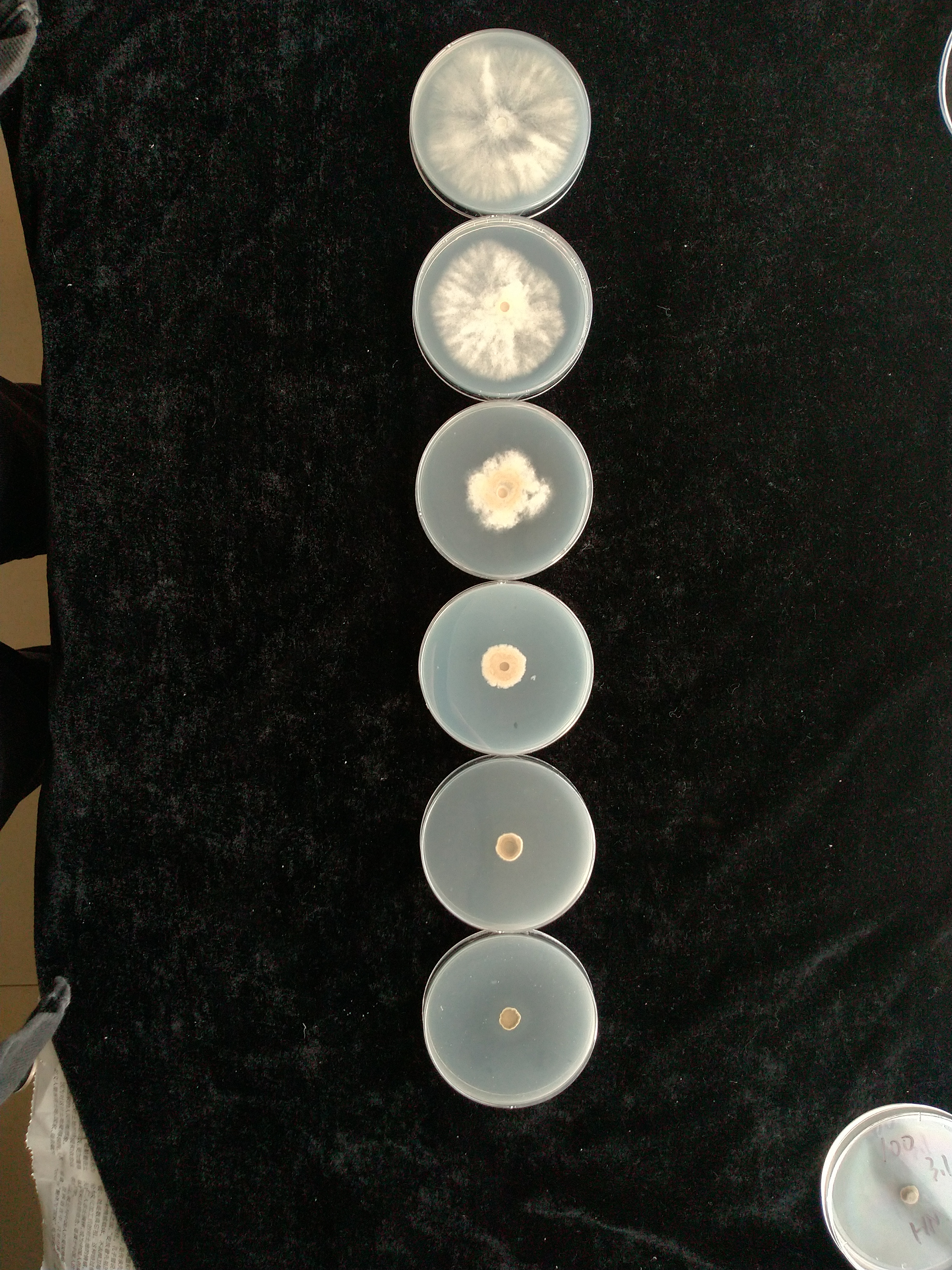

Supplement: Supplemental Information 10 [file peerj-08-9626-s010.zip › EC50valuas/thymol2.jpg]

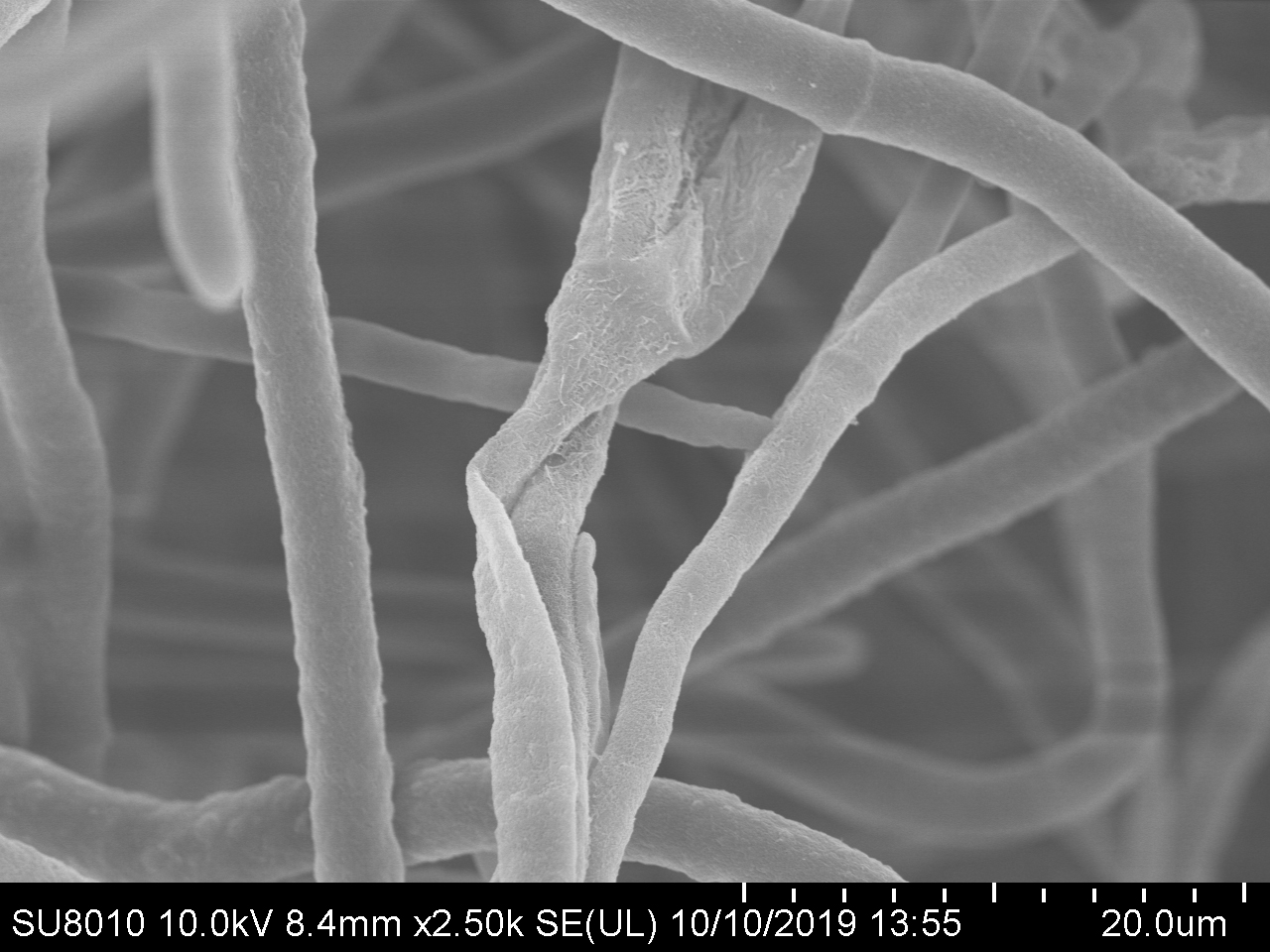

Supplement: Supplemental Information 11 [file peerj-08-9626-s011.zip › SEM images/Carvacrol.jpg]

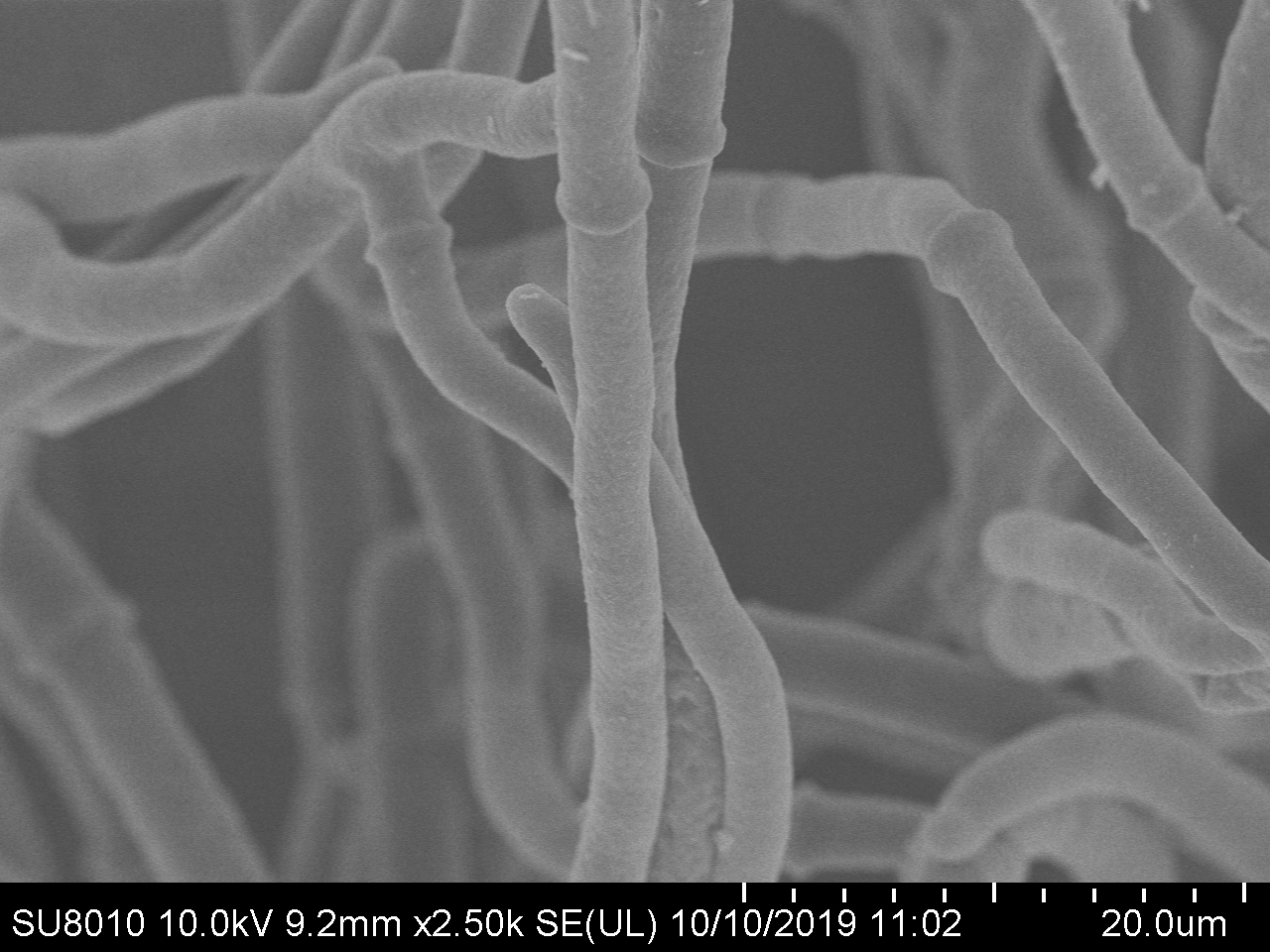

Supplement: Supplemental Information 11 [file peerj-08-9626-s011.zip › SEM images/Control.jpg]

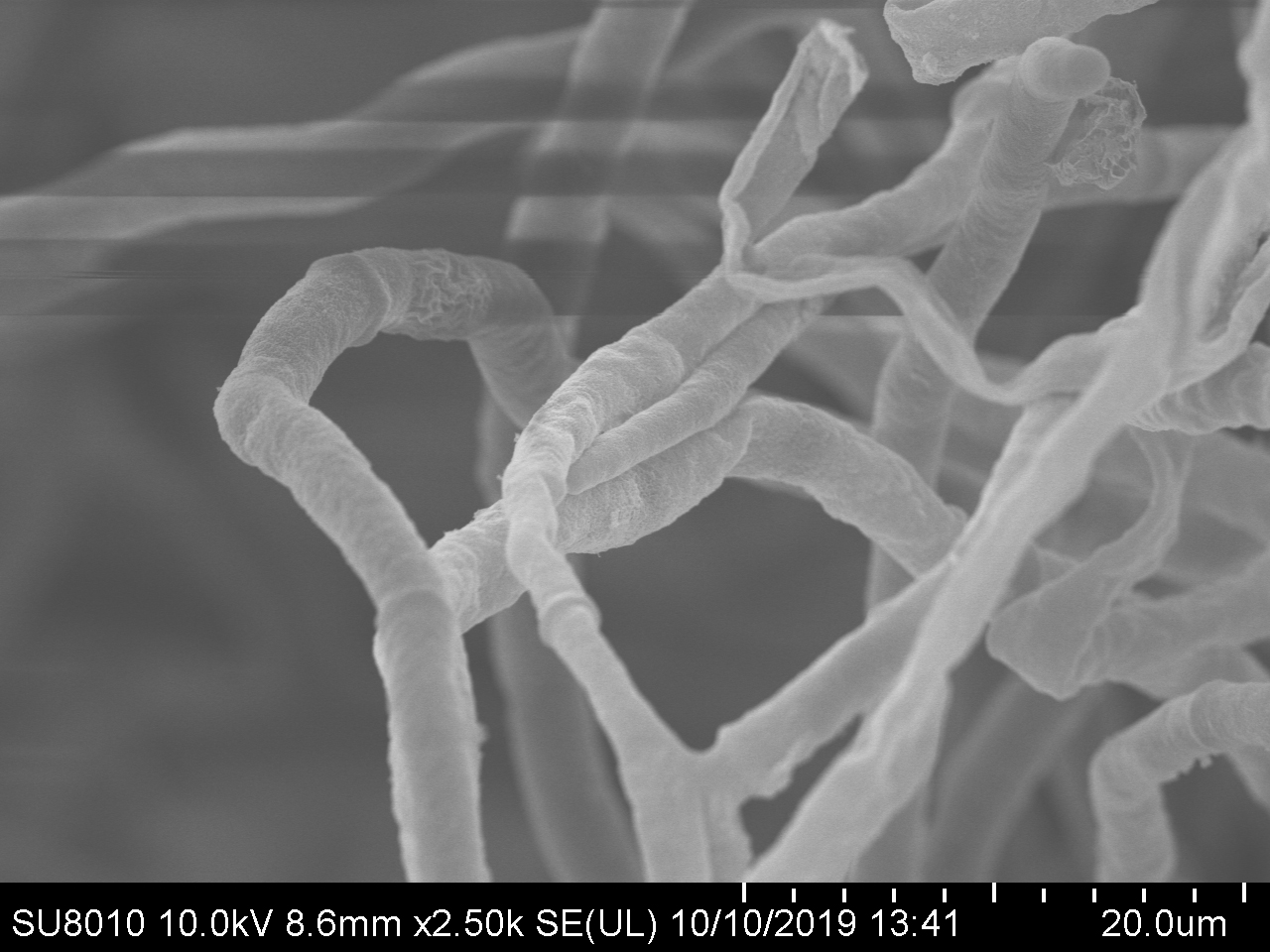

Supplement: Supplemental Information 11 [file peerj-08-9626-s011.zip › SEM images/Thymol.jpg]

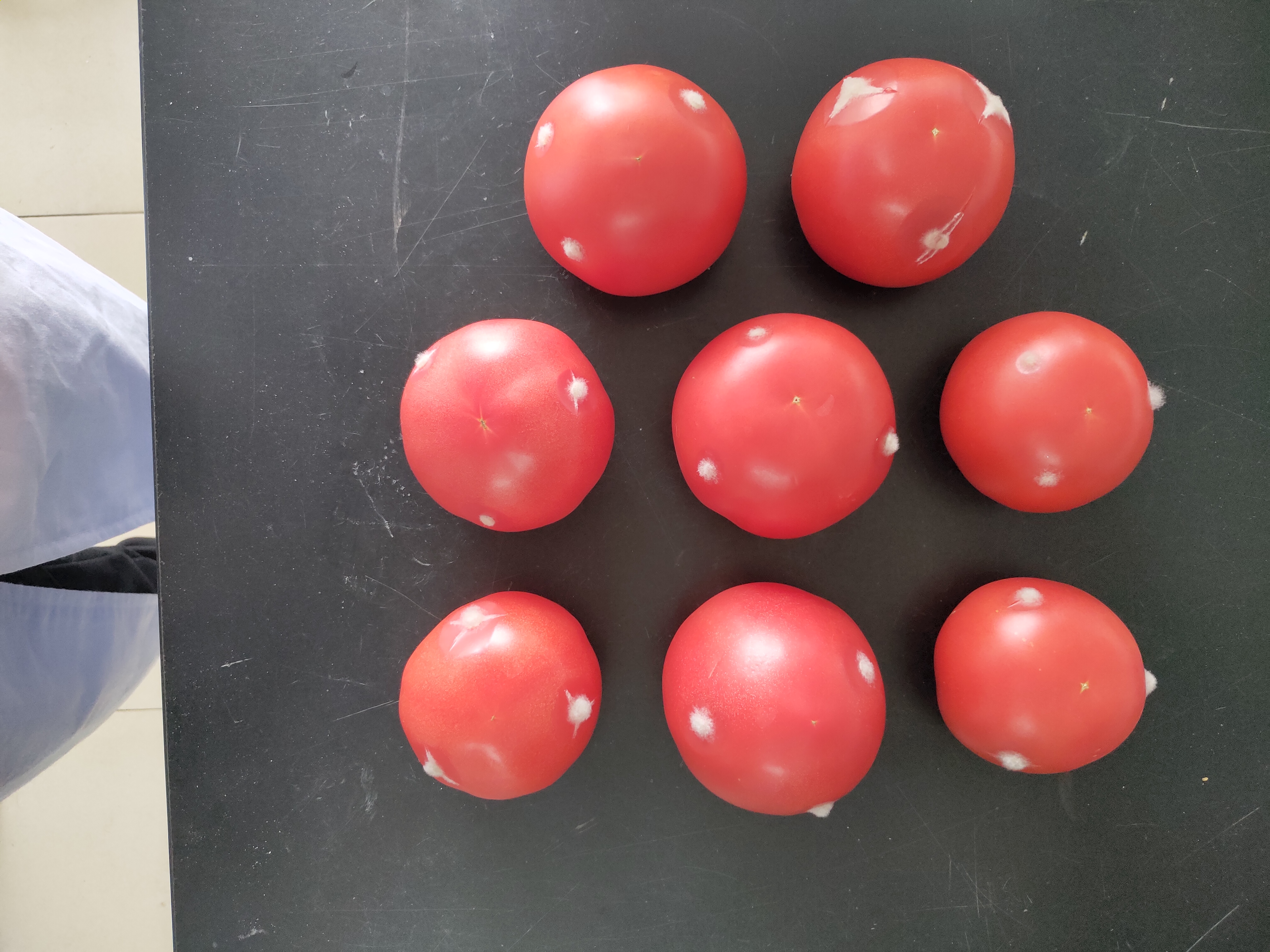

Supplement: Supplemental Information 12 [file peerj-08-9626-s012.zip › protective and therapeutic effects/protective effect.jpg]

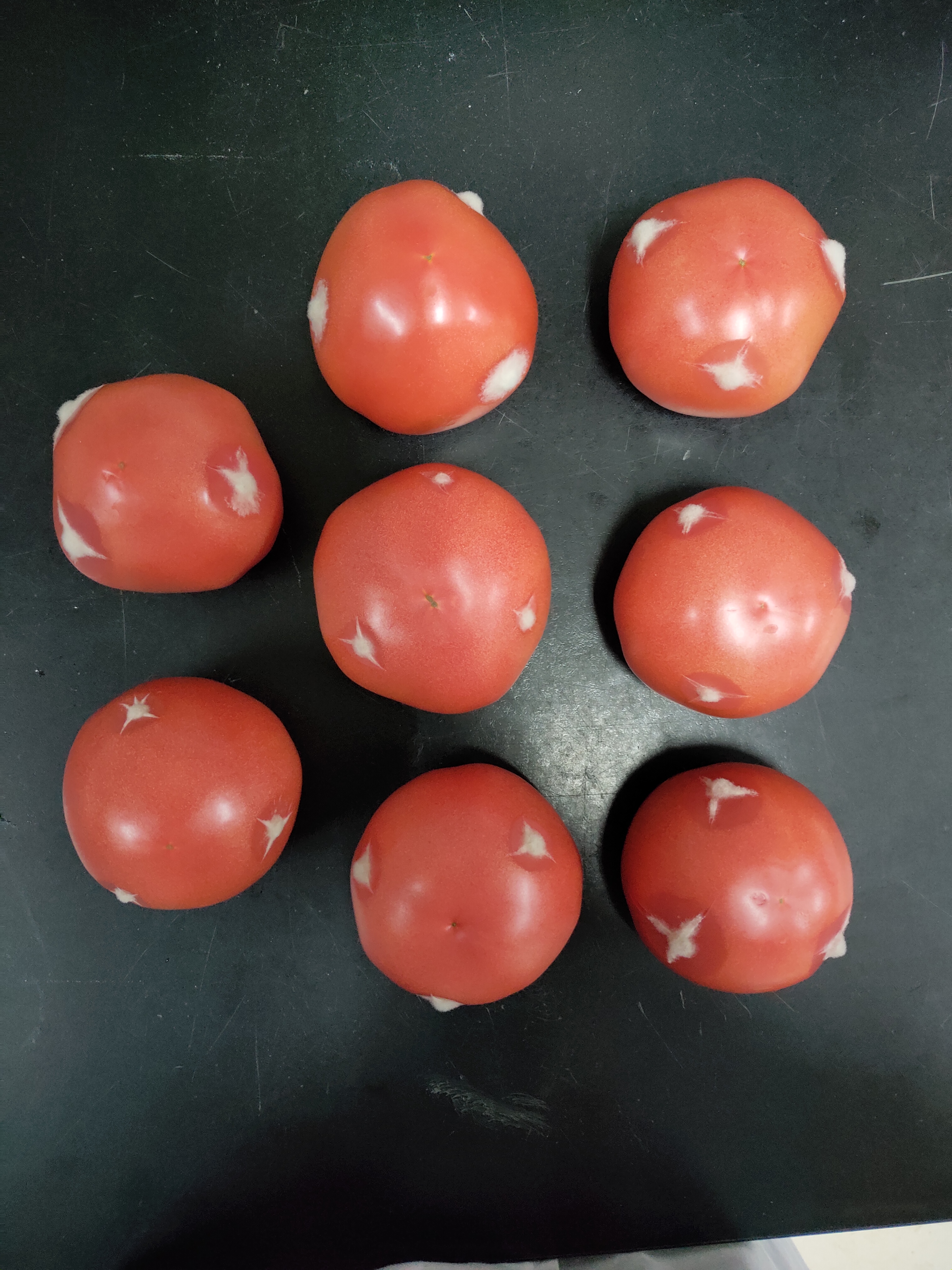

Supplement: Supplemental Information 12 [file peerj-08-9626-s012.zip › protective and therapeutic effects/therapeutic effect.jpg]

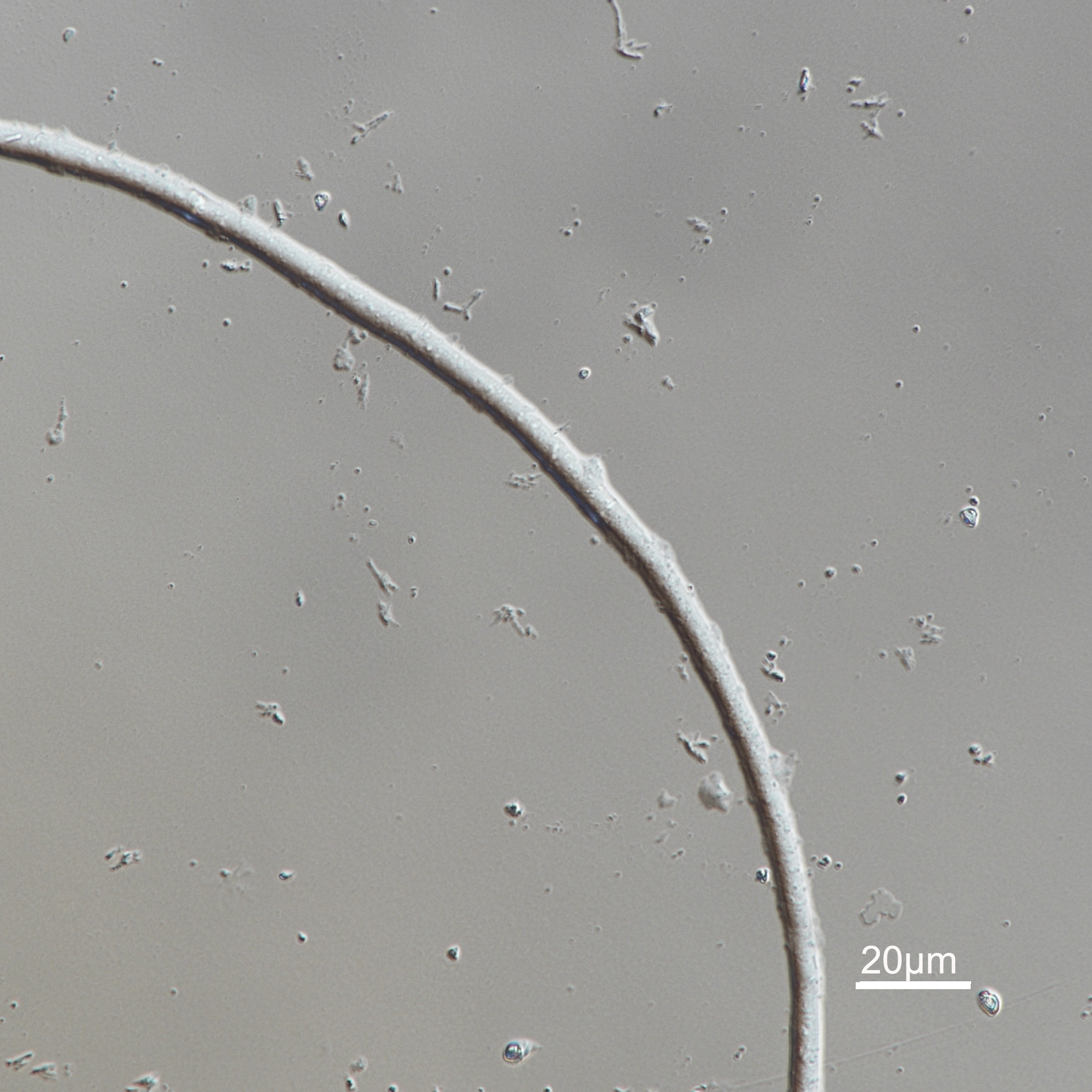

Supplement: Supplemental Information 13 [file peerj-08-9626-s013.zip › ROS/carvacrol bright field.jpg]

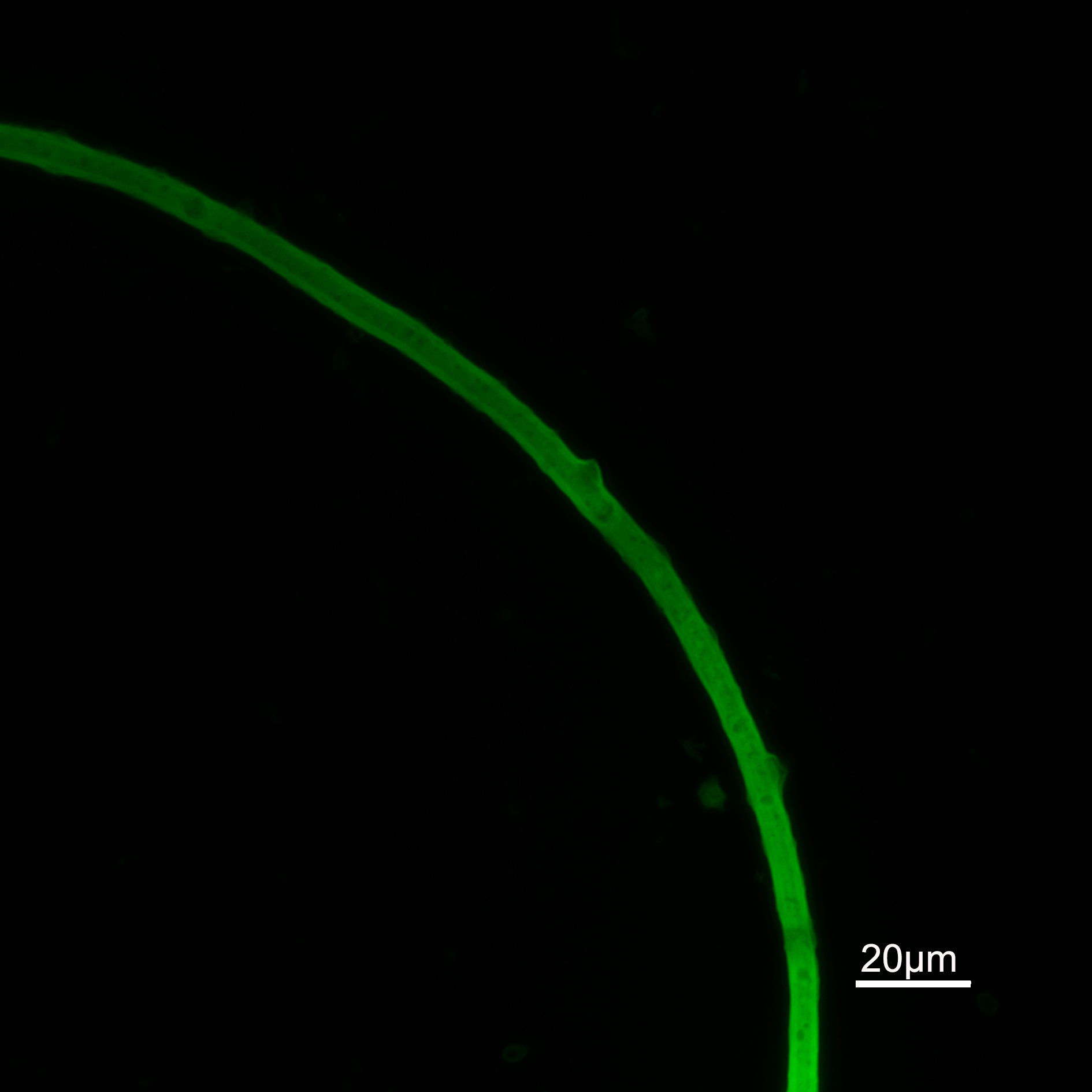

Supplement: Supplemental Information 13 [file peerj-08-9626-s013.zip › ROS/carvacrol with H2DCFDA.jpg]

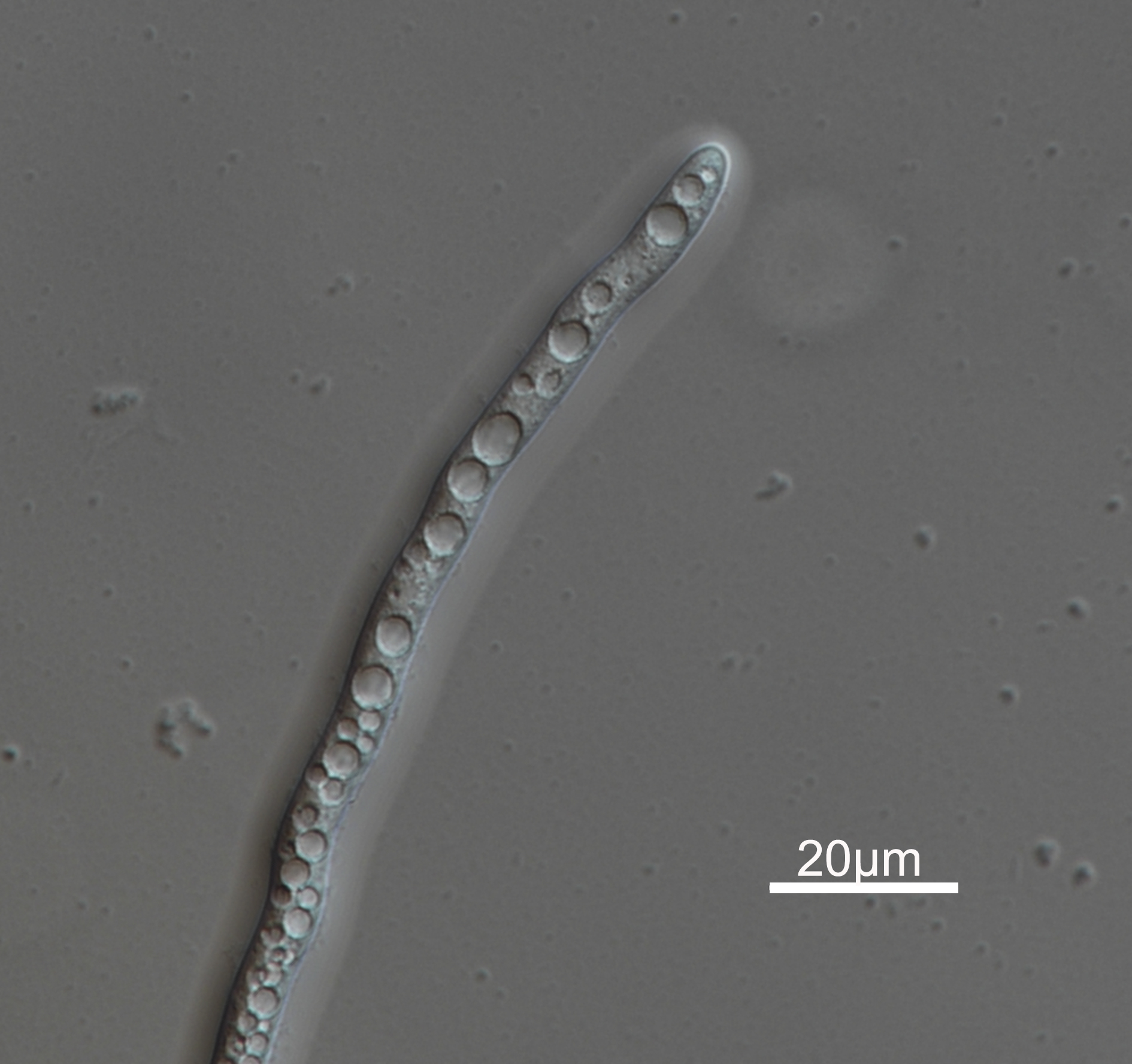

Supplement: Supplemental Information 13 [file peerj-08-9626-s013.zip › ROS/contol bright field.jpg]

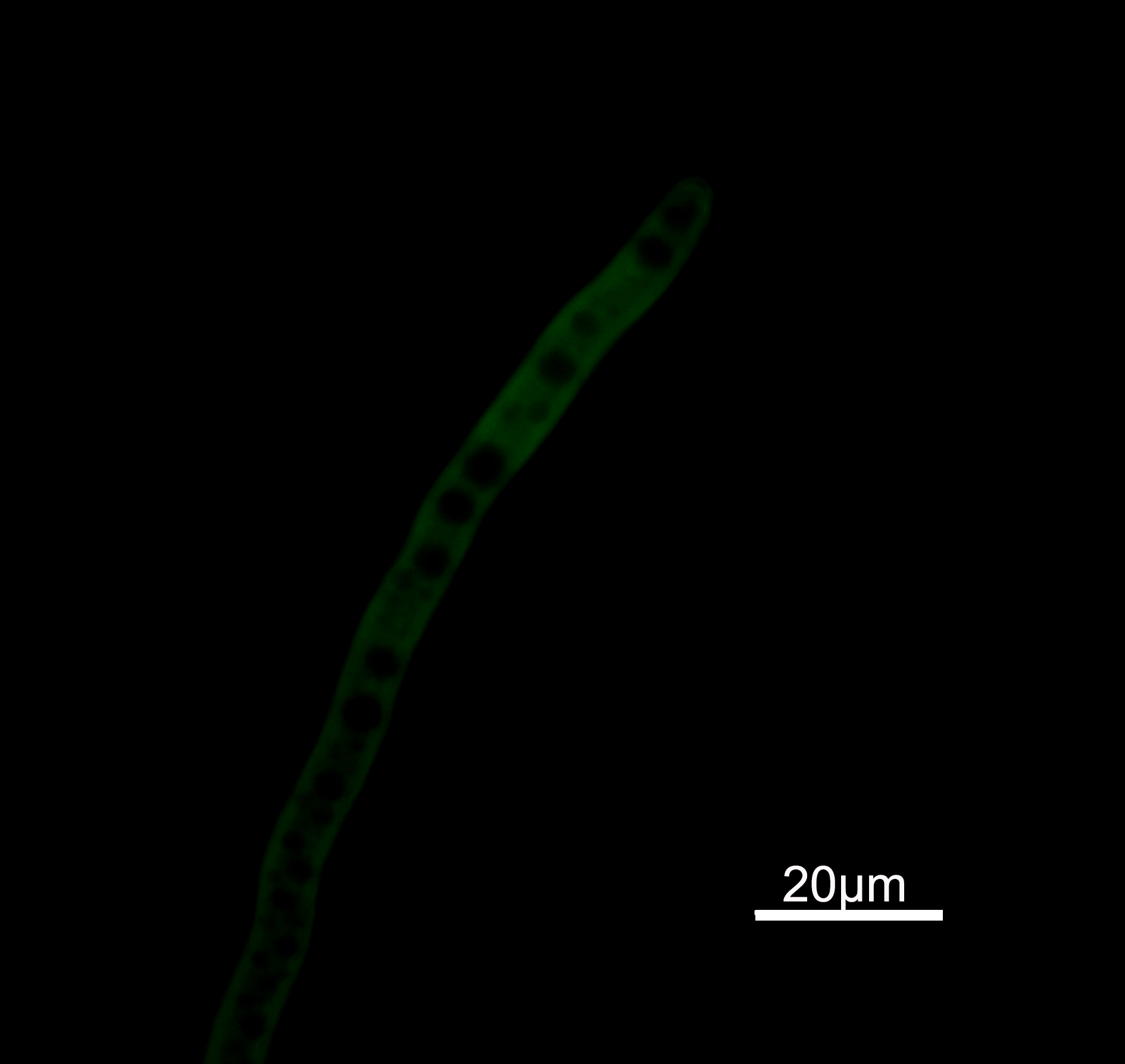

Supplement: Supplemental Information 13 [file peerj-08-9626-s013.zip › ROS/control with H2DCFDA.jpg]

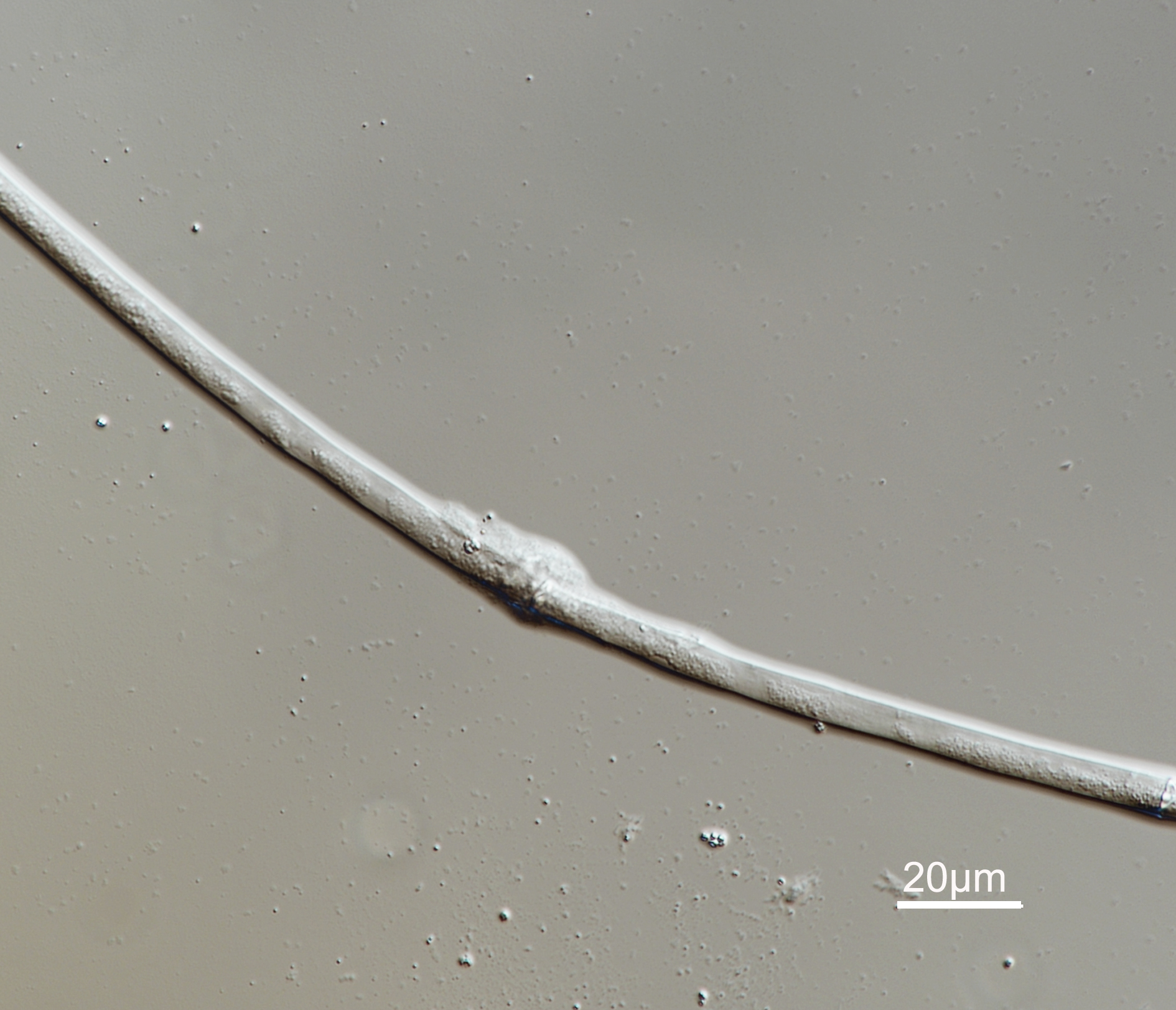

Supplement: Supplemental Information 13 [file peerj-08-9626-s013.zip › ROS/thymol bright field.jpg]

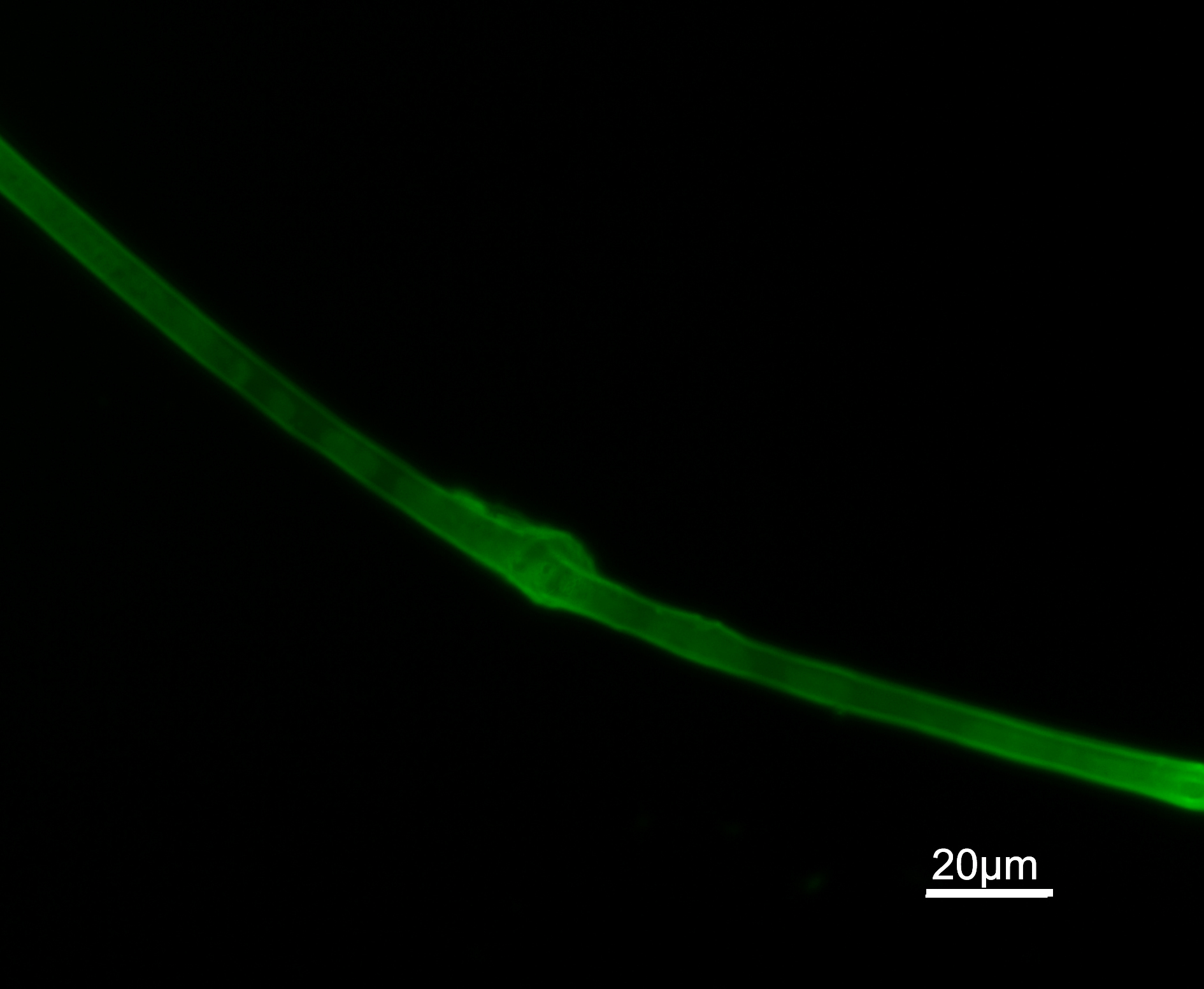

Supplement: Supplemental Information 13 [file peerj-08-9626-s013.zip › ROS/thymol with H2DCFDA.jpg]

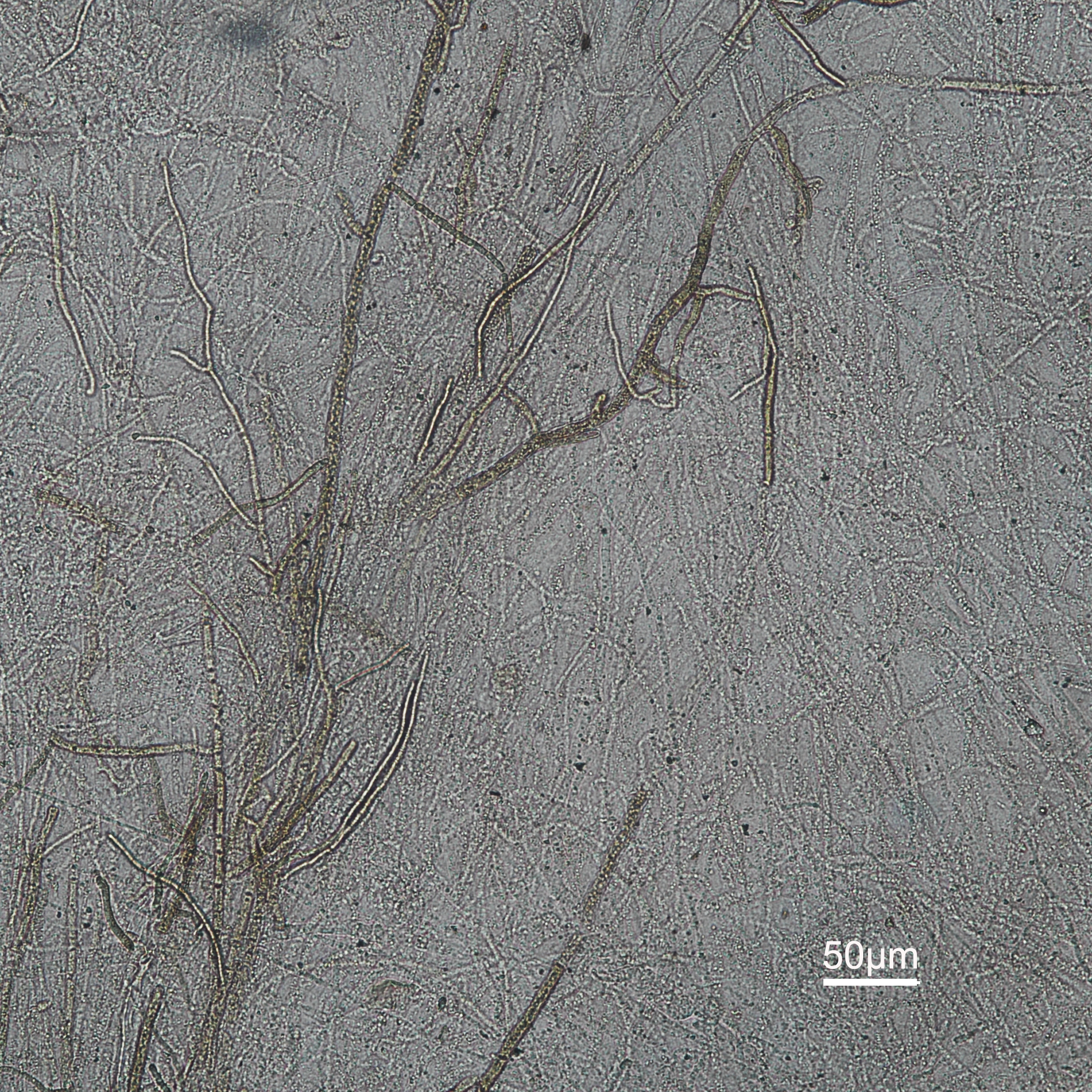

Supplement: Supplemental Information 14 [file peerj-08-9626-s014.zip › mitochondrial injury/carvacrol bright field.jpg]

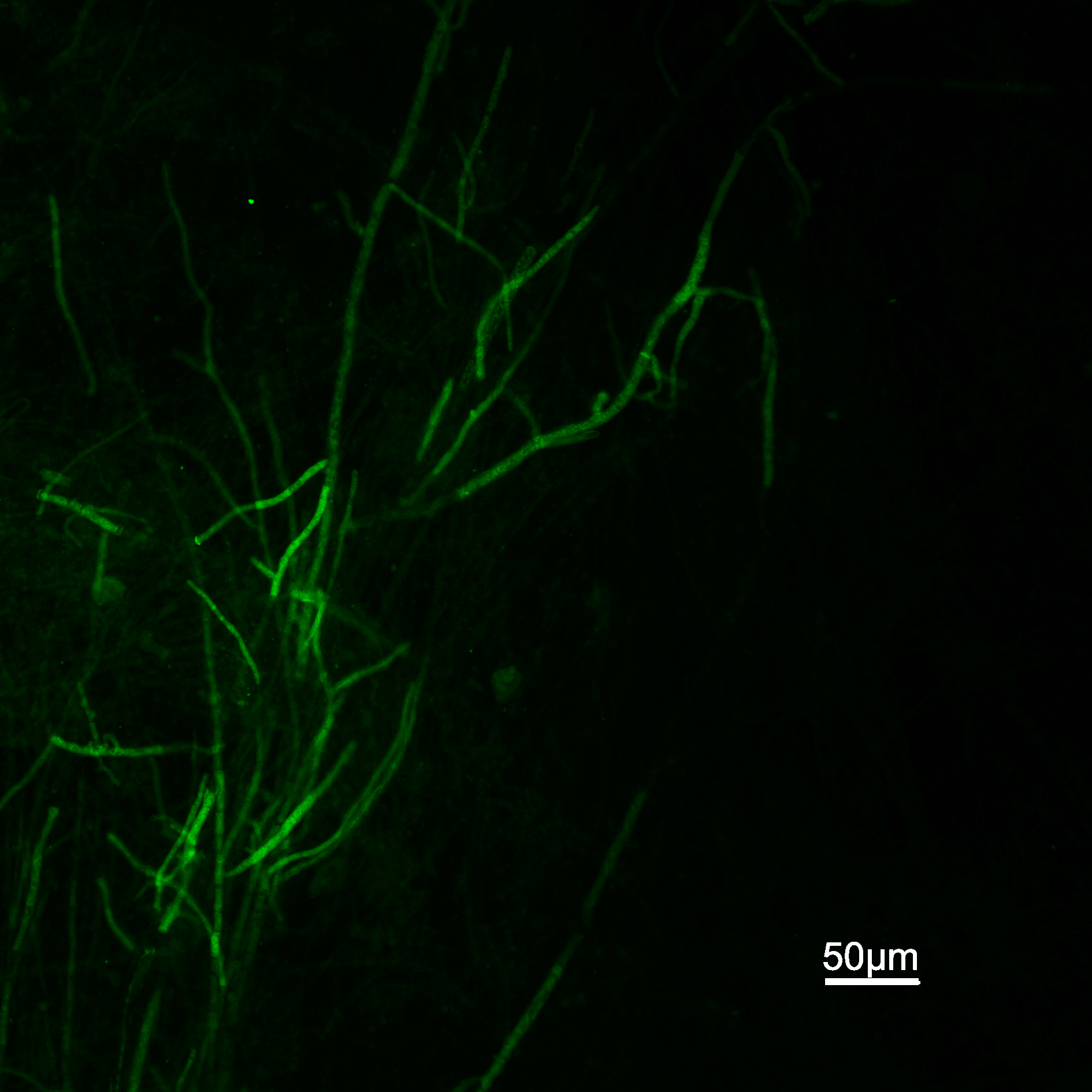

Supplement: Supplemental Information 14 [file peerj-08-9626-s014.zip › mitochondrial injury/carvacrol with Rhodamine 123.jpg]

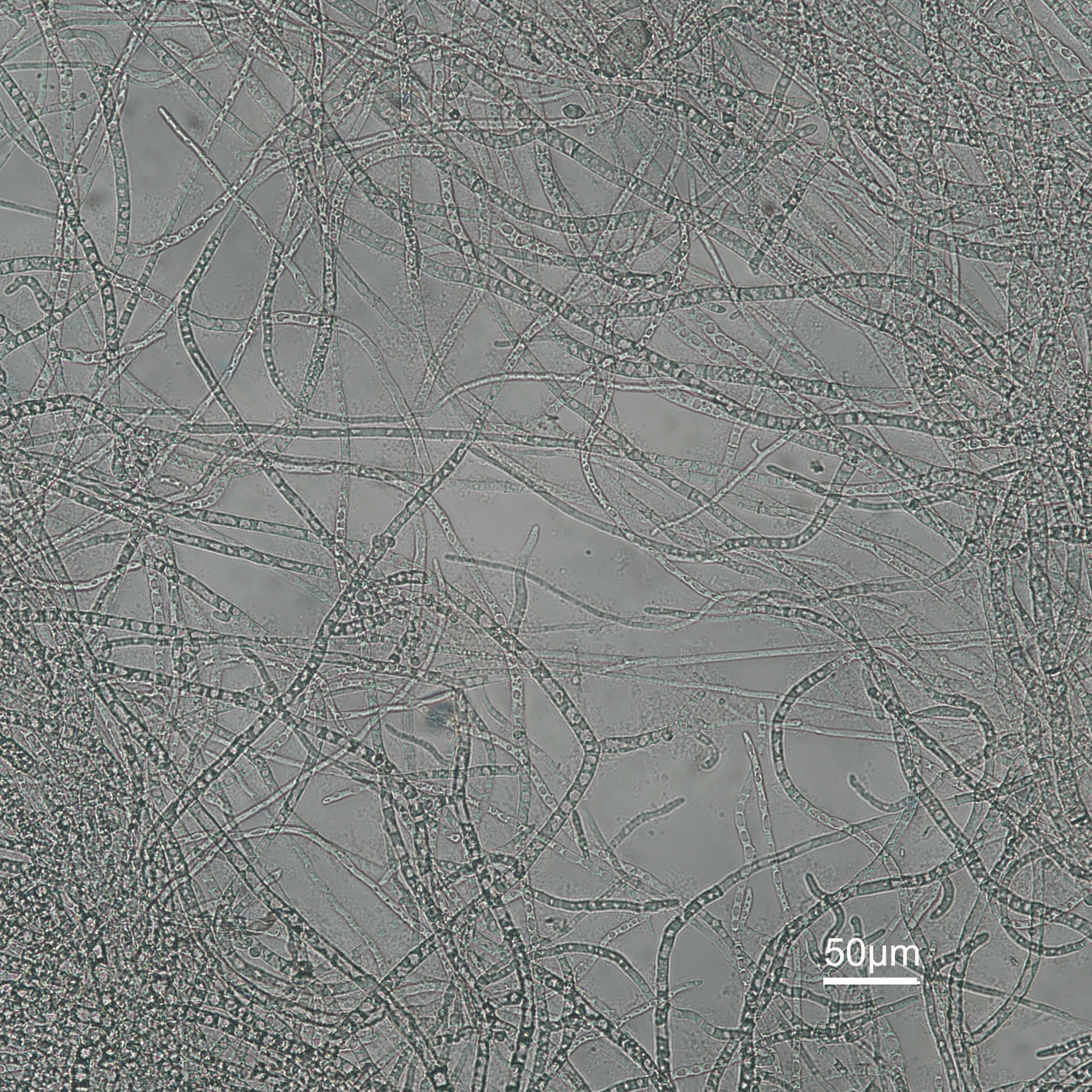

Supplement: Supplemental Information 14 [file peerj-08-9626-s014.zip › mitochondrial injury/control bright field.jpg]

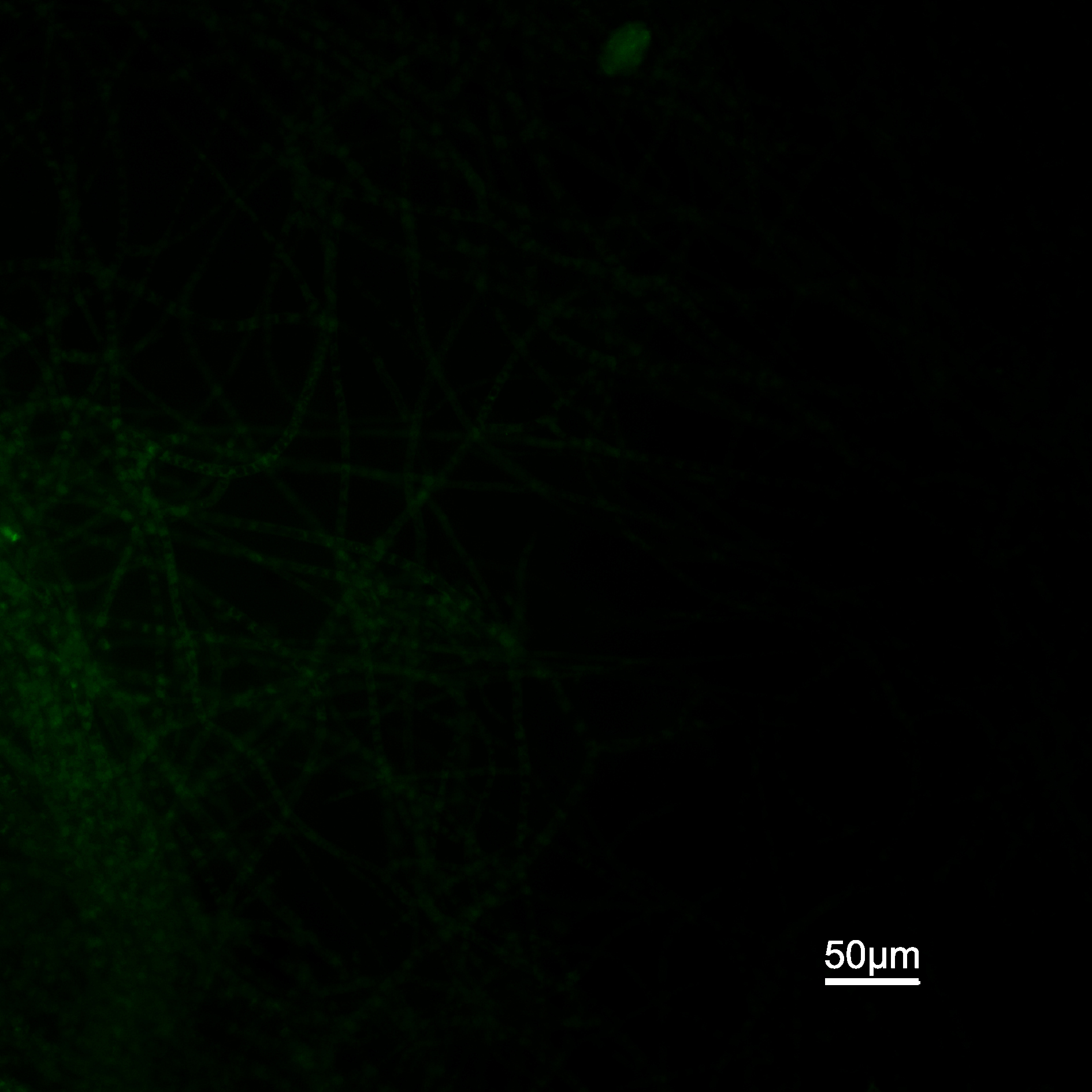

Supplement: Supplemental Information 14 [file peerj-08-9626-s014.zip › mitochondrial injury/control with Rhodamine 123.jpg]

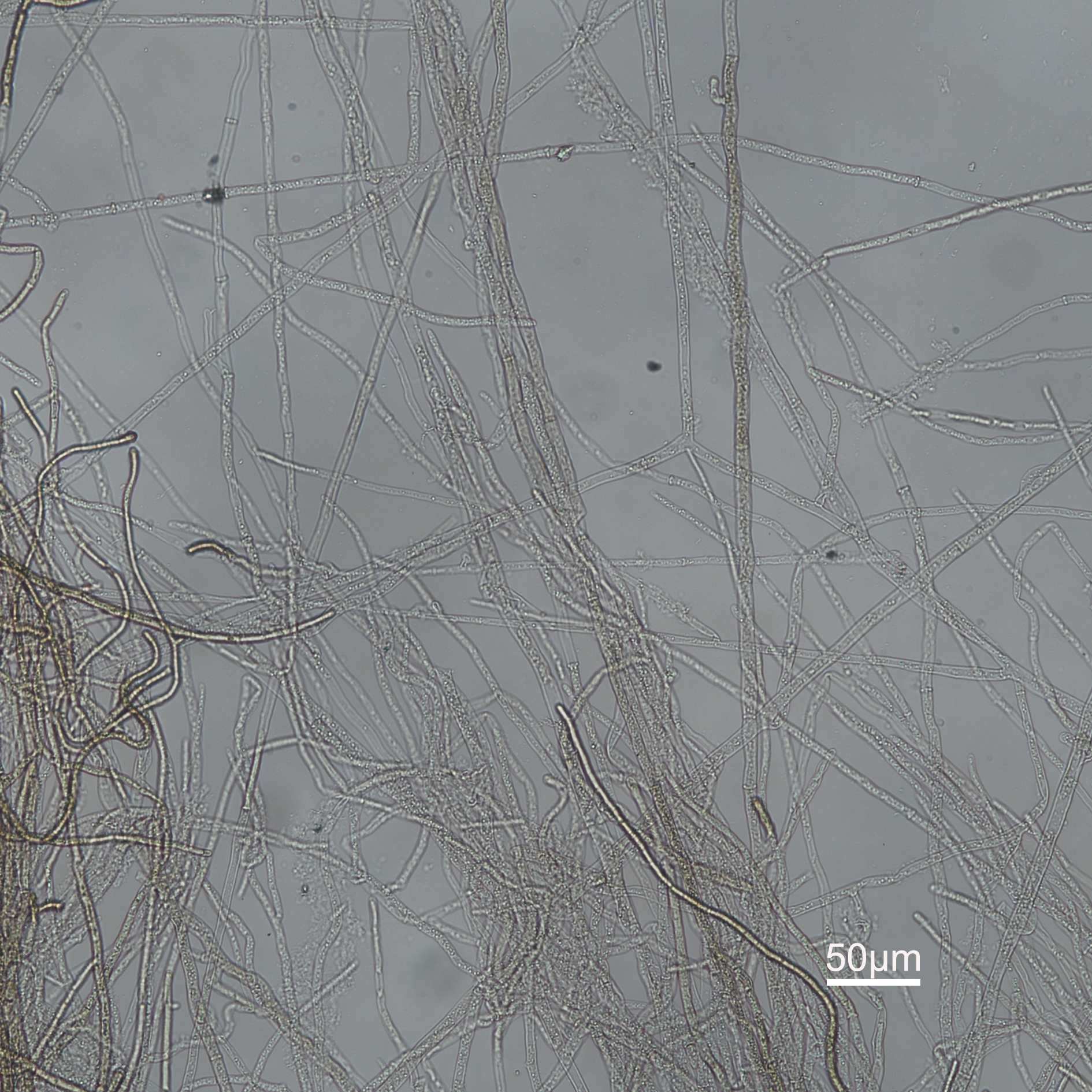

Supplement: Supplemental Information 14 [file peerj-08-9626-s014.zip › mitochondrial injury/thymol bright field.jpg]

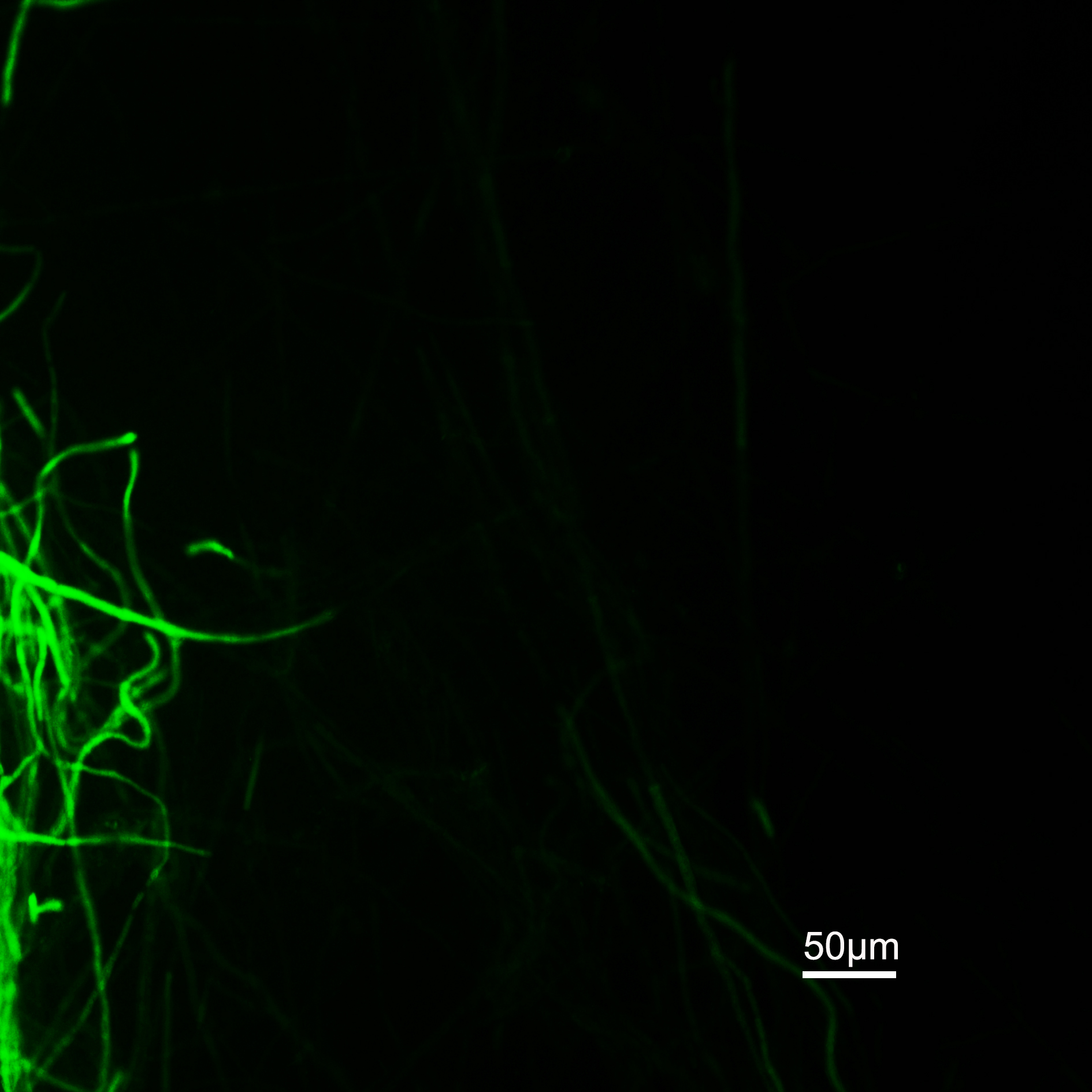

Supplement: Supplemental Information 14 [file peerj-08-9626-s014.zip › mitochondrial injury/thymol with Rhodamine 123.jpg]

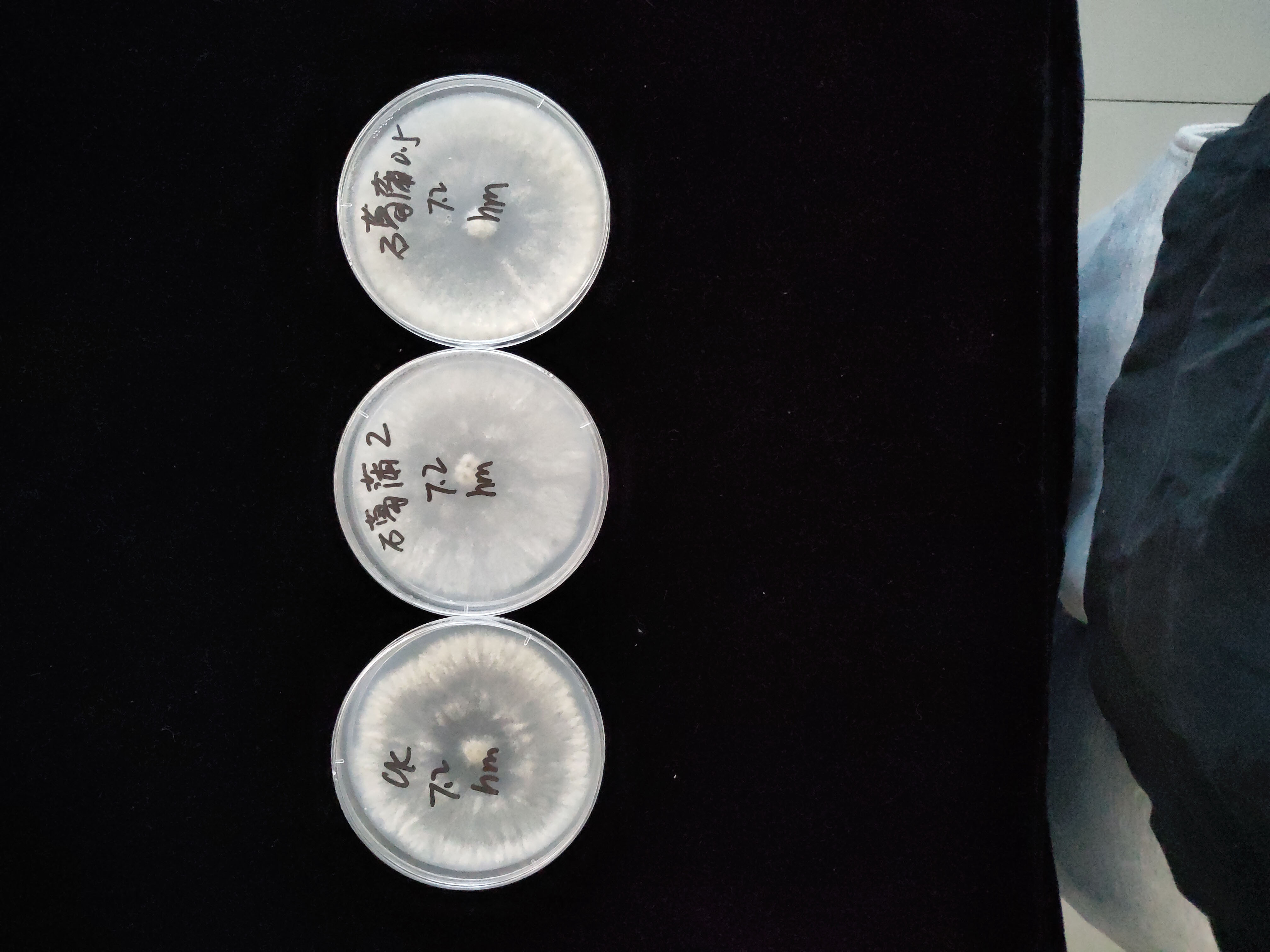

Supplement: Supplemental Information 15 [file peerj-08-9626-s015.zip › Inhibitory activities of 17 plant essential oils on mycelium growth of B. cinerea/photos/Acorus tatarinowii.jpg]

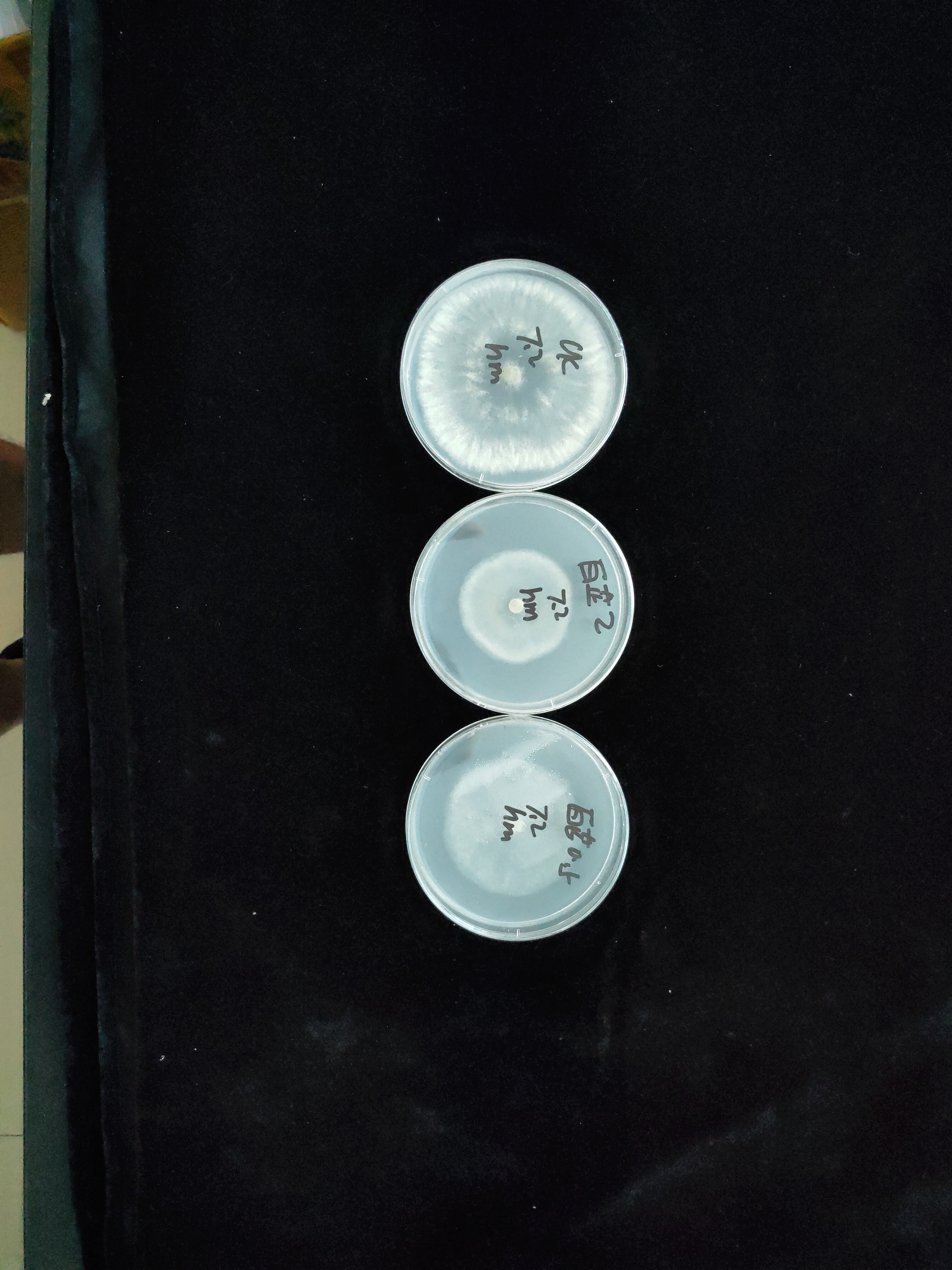

Supplement: Supplemental Information 15 [file peerj-08-9626-s015.zip › Inhibitory activities of 17 plant essential oils on mycelium growth of B. cinerea/photos/Angelica dahurica.jpg]

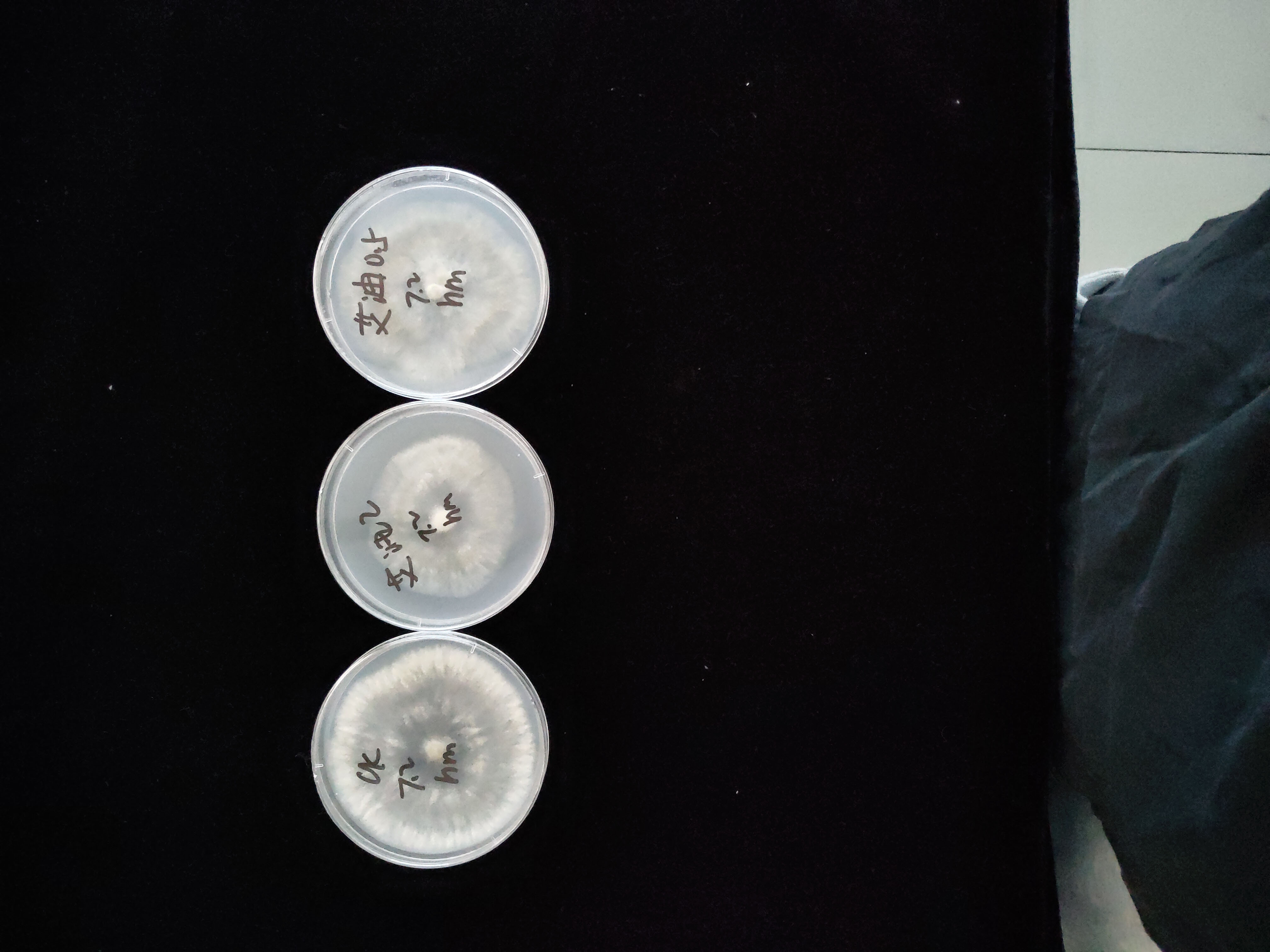

Supplement: Supplemental Information 15 [file peerj-08-9626-s015.zip › Inhibitory activities of 17 plant essential oils on mycelium growth of B. cinerea/photos/Artemisia argyi.jpg]

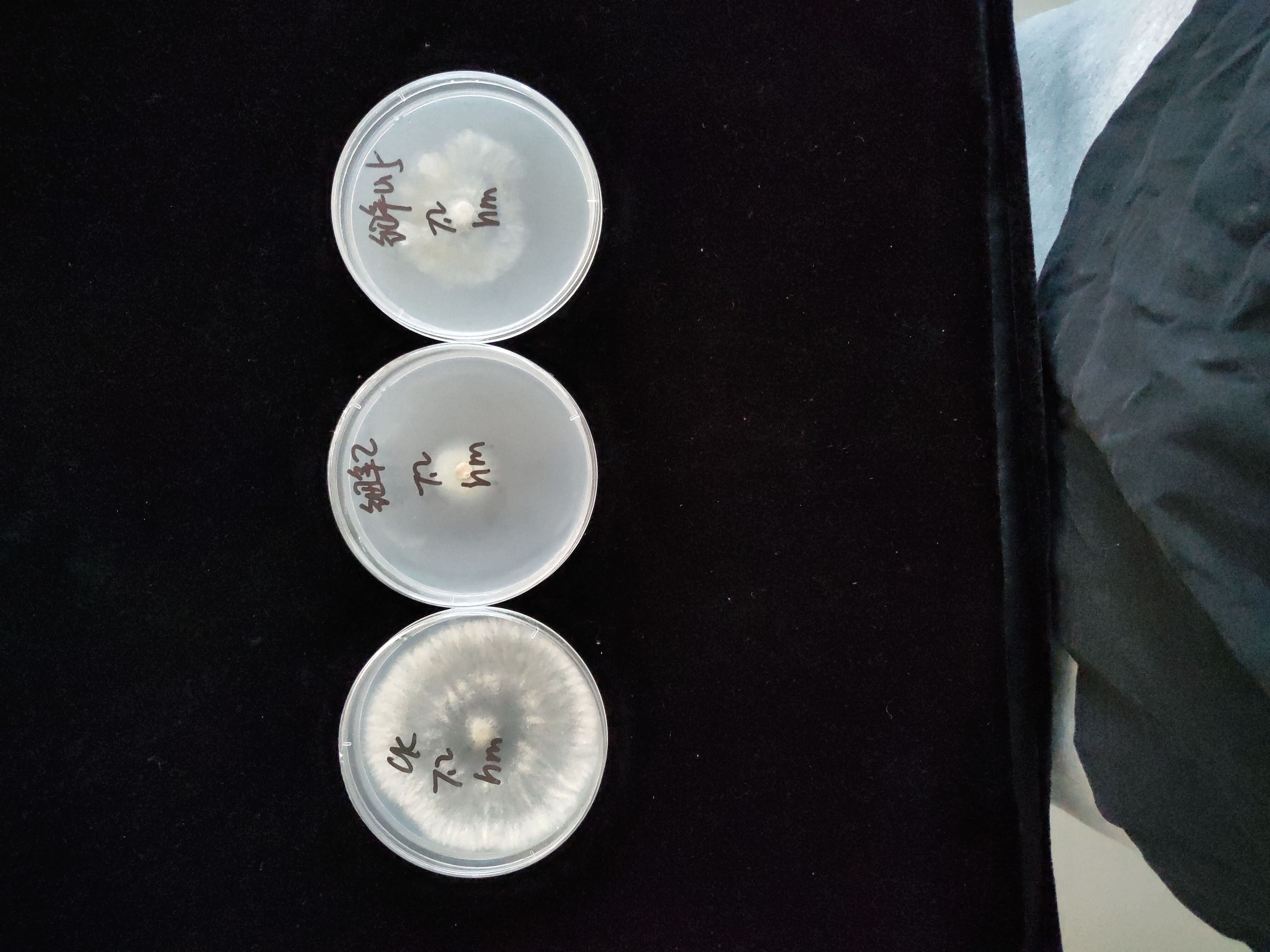

Supplement: Supplemental Information 15 [file peerj-08-9626-s015.zip › Inhibitory activities of 17 plant essential oils on mycelium growth of B. cinerea/photos/Asarum sieboldii.jpg]

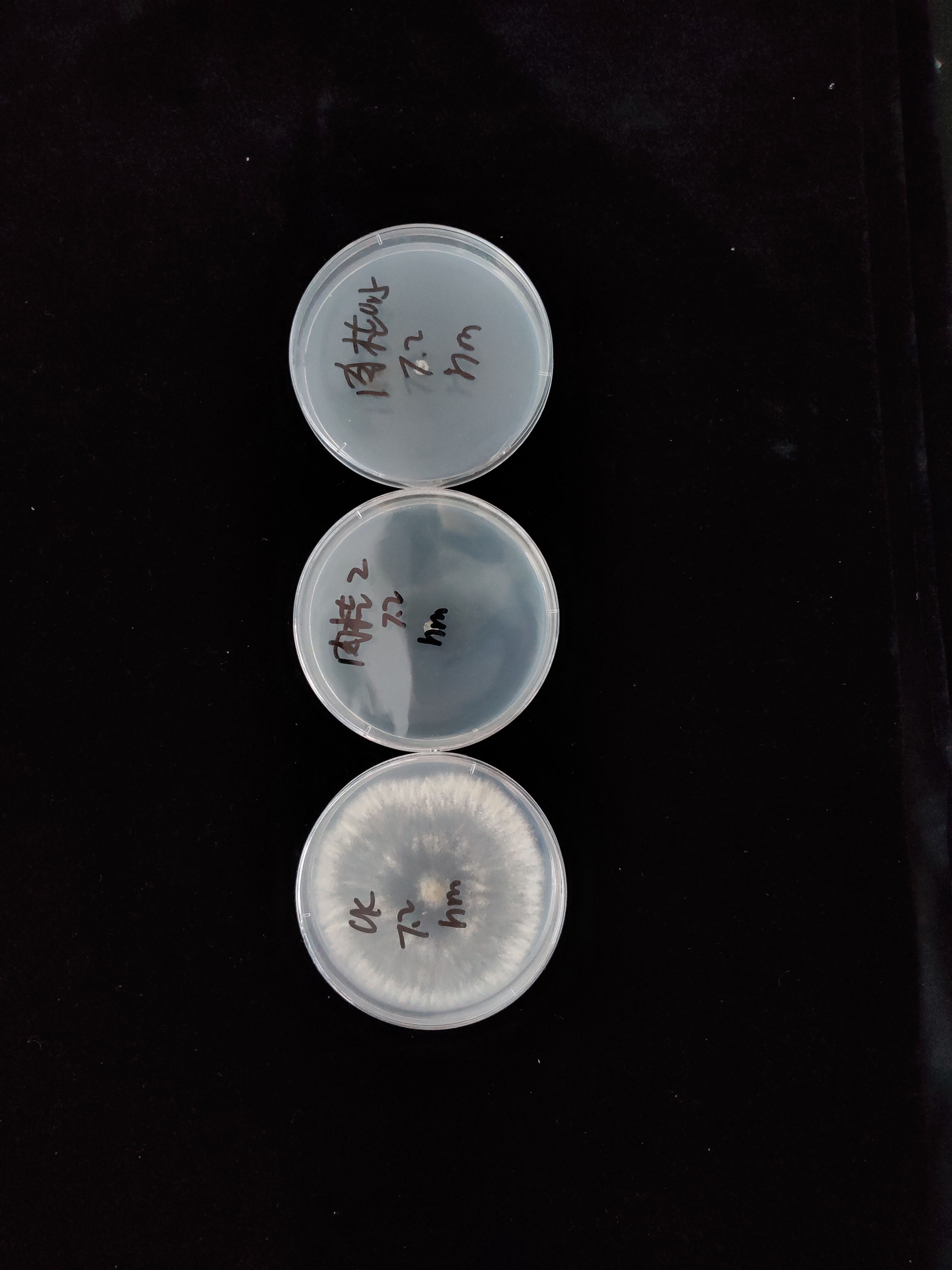

Supplement: Supplemental Information 15 [file peerj-08-9626-s015.zip › Inhibitory activities of 17 plant essential oils on mycelium growth of B. cinerea/photos/Cinnamomum cassia.jpg]

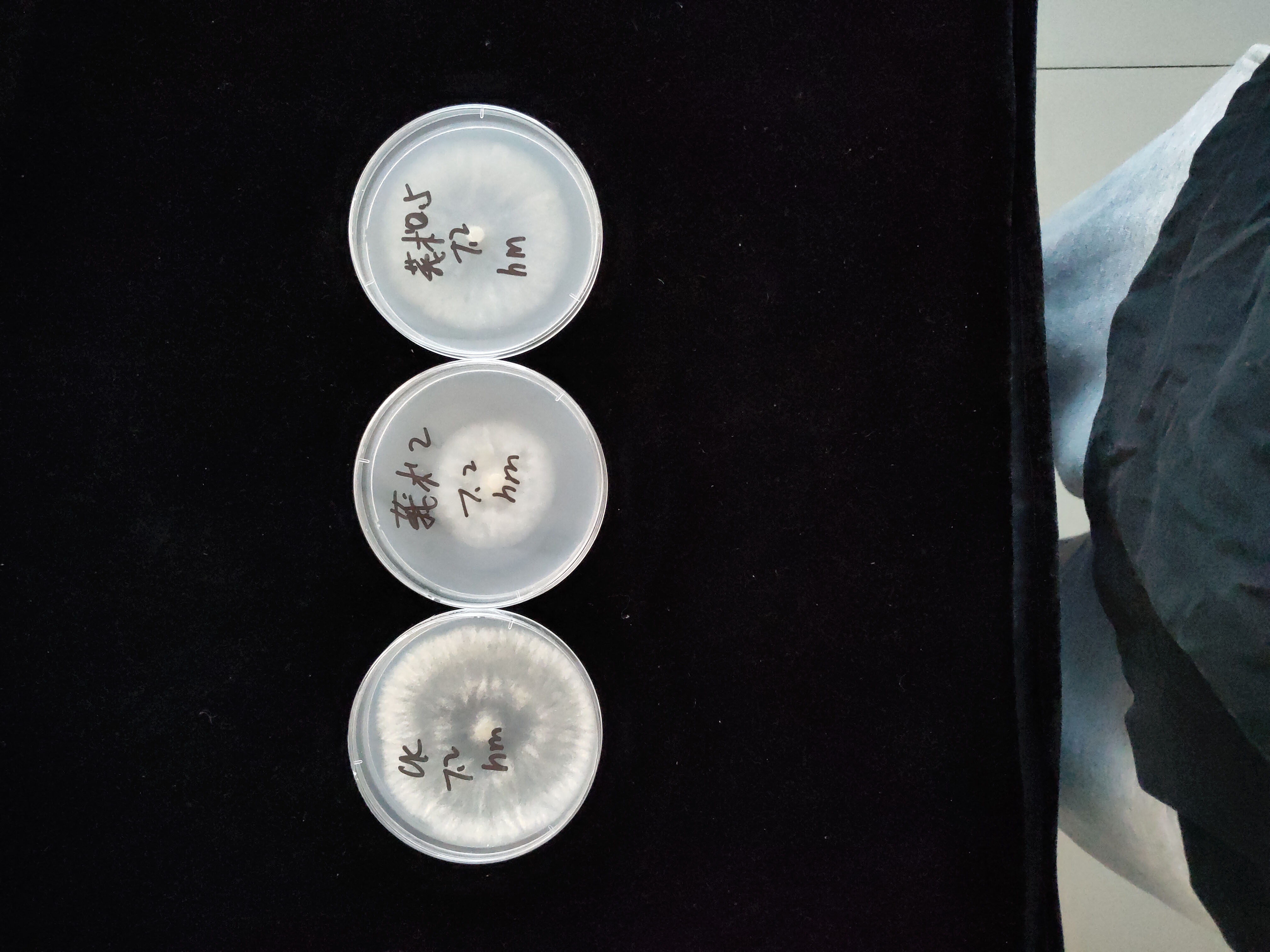

Supplement: Supplemental Information 15 [file peerj-08-9626-s015.zip › Inhibitory activities of 17 plant essential oils on mycelium growth of B. cinerea/photos/Curcuma zedoaria.jpg]

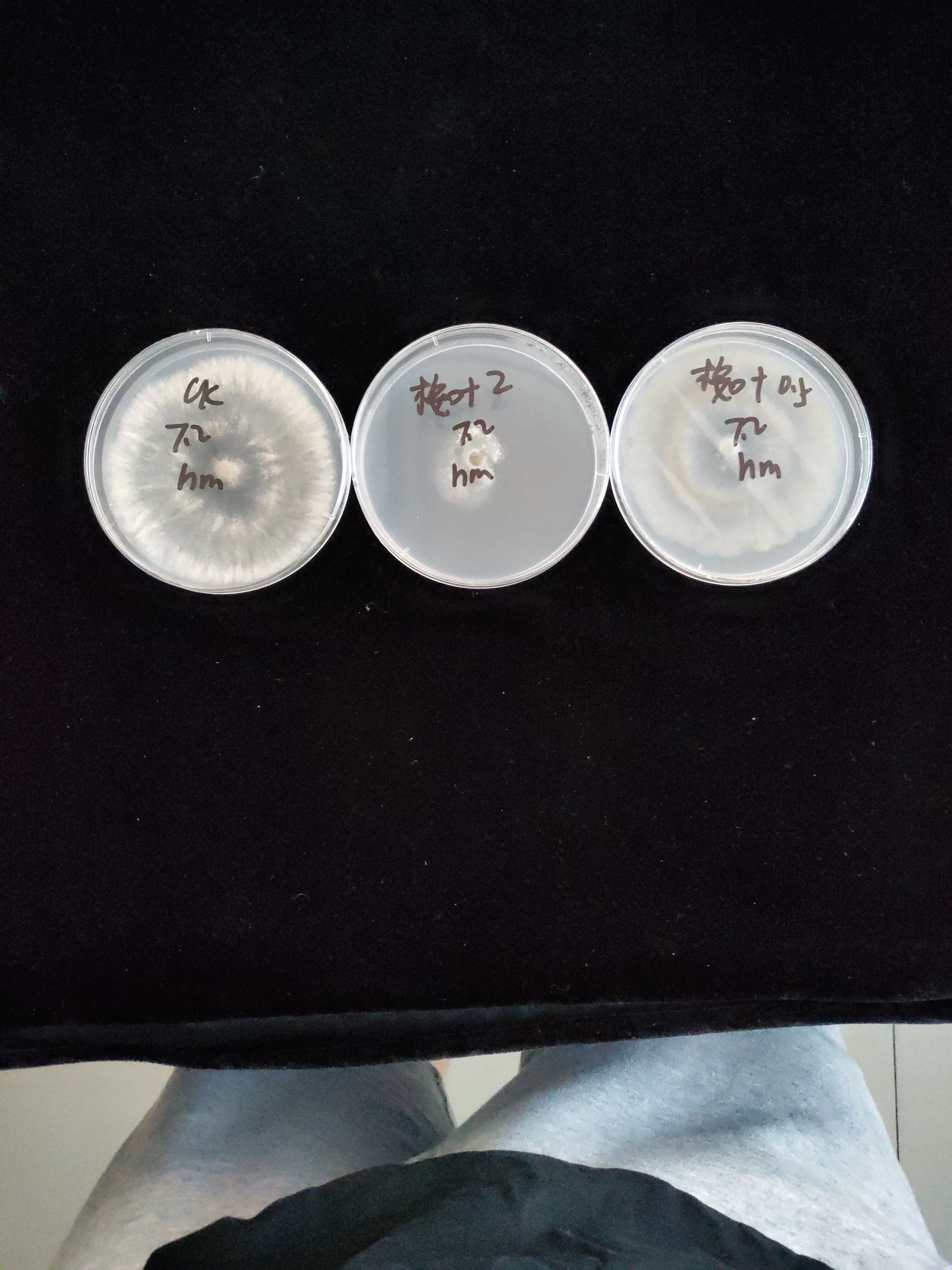

Supplement: Supplemental Information 15 [file peerj-08-9626-s015.zip › Inhibitory activities of 17 plant essential oils on mycelium growth of B. cinerea/photos/Eucalyptus globulus.jpg]

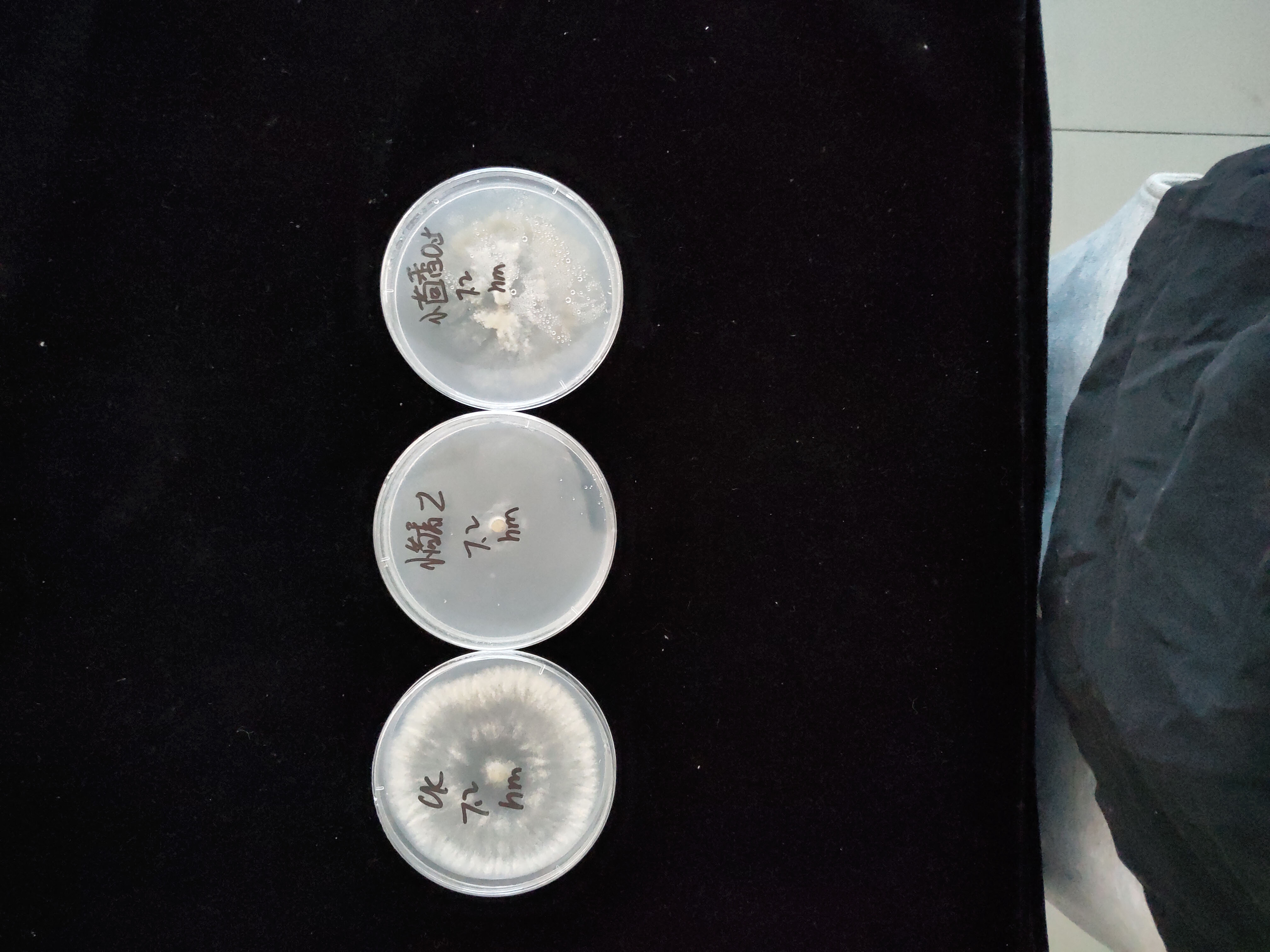

Supplement: Supplemental Information 15 [file peerj-08-9626-s015.zip › Inhibitory activities of 17 plant essential oils on mycelium growth of B. cinerea/photos/Foeniculum vulgare.jpg]

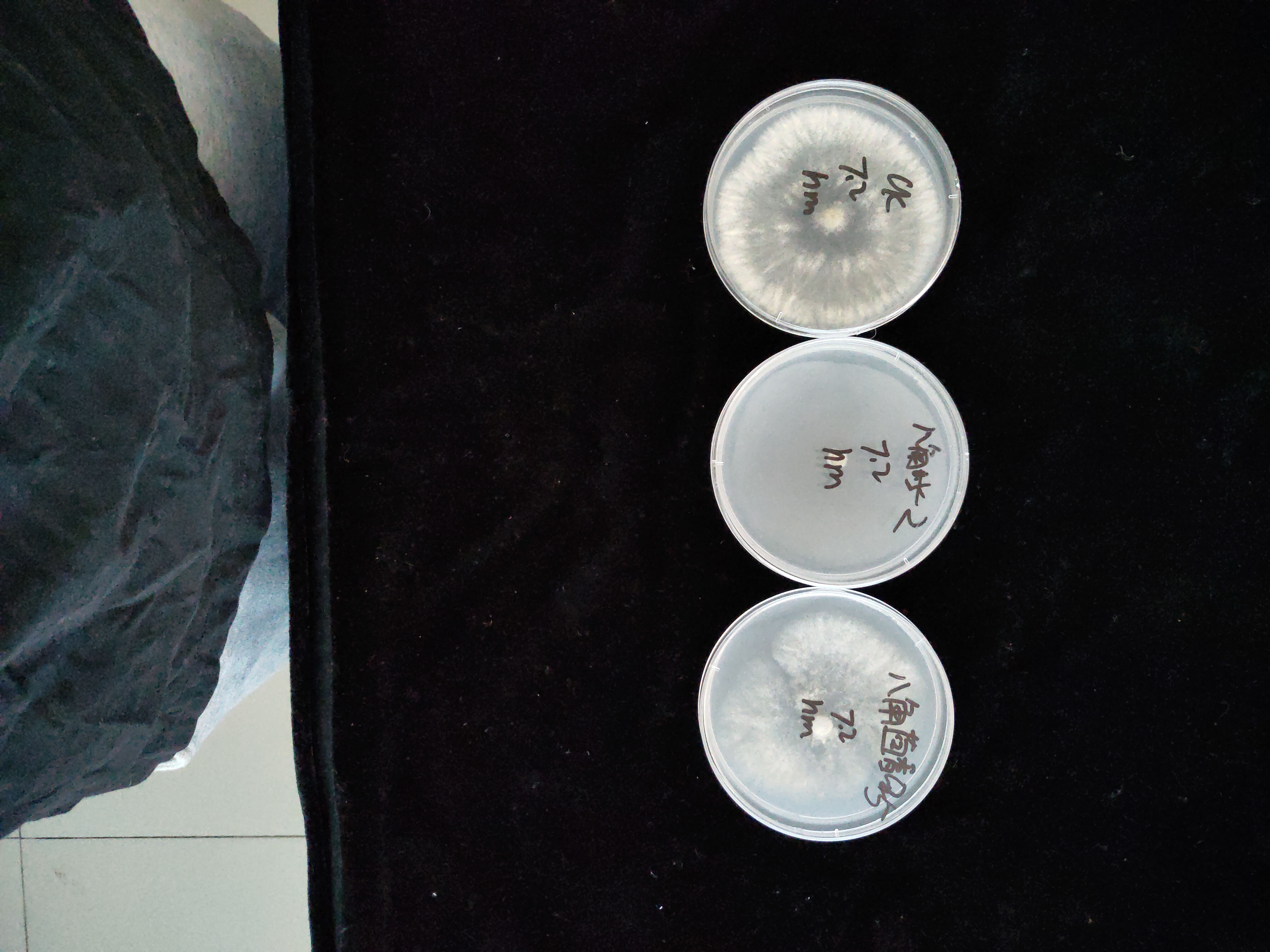

Supplement: Supplemental Information 15 [file peerj-08-9626-s015.zip › Inhibitory activities of 17 plant essential oils on mycelium growth of B. cinerea/photos/Illicium verum.jpg]

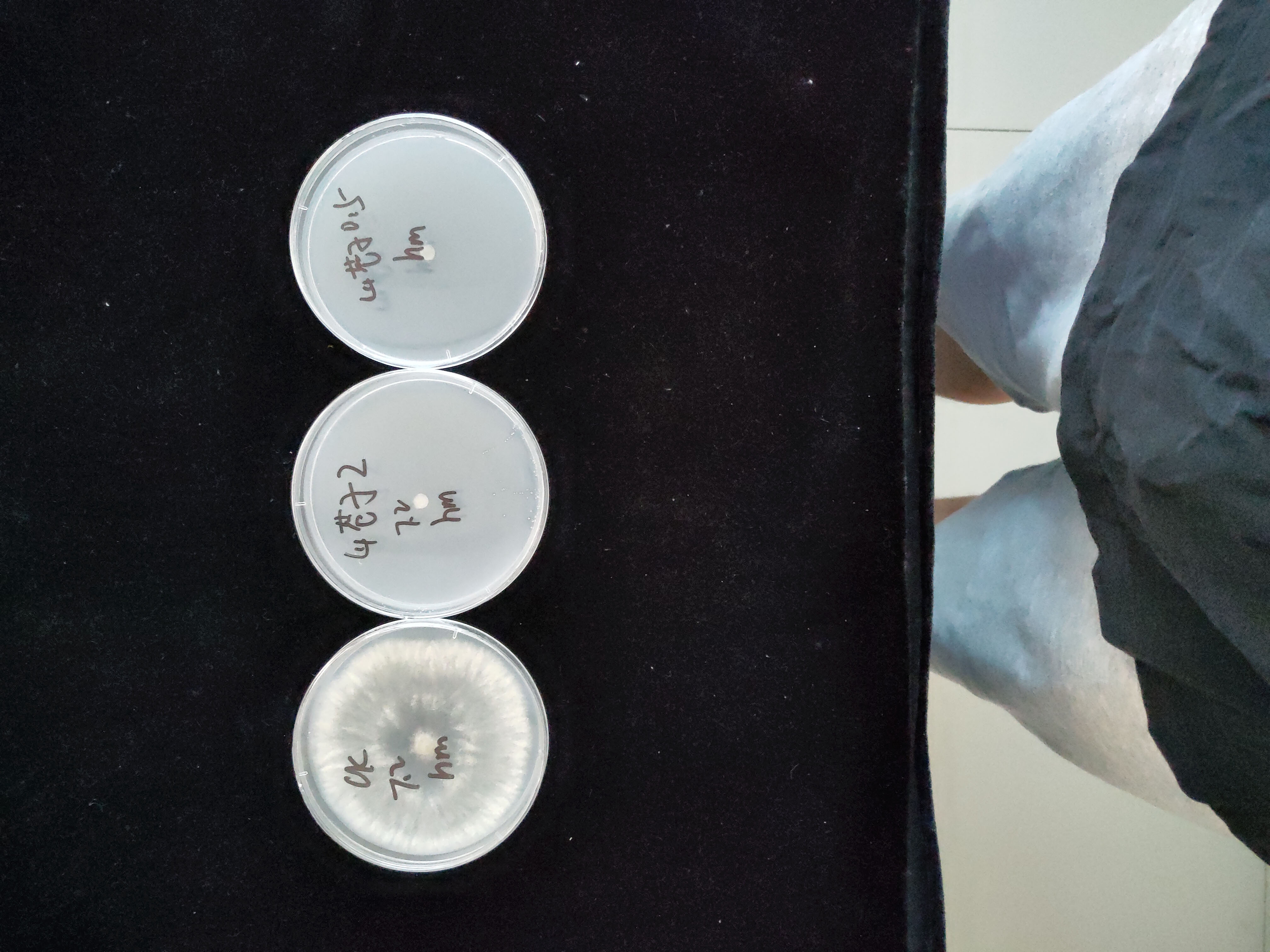

Supplement: Supplemental Information 15 [file peerj-08-9626-s015.zip › Inhibitory activities of 17 plant essential oils on mycelium growth of B. cinerea/photos/Litsea cubeba.jpg]

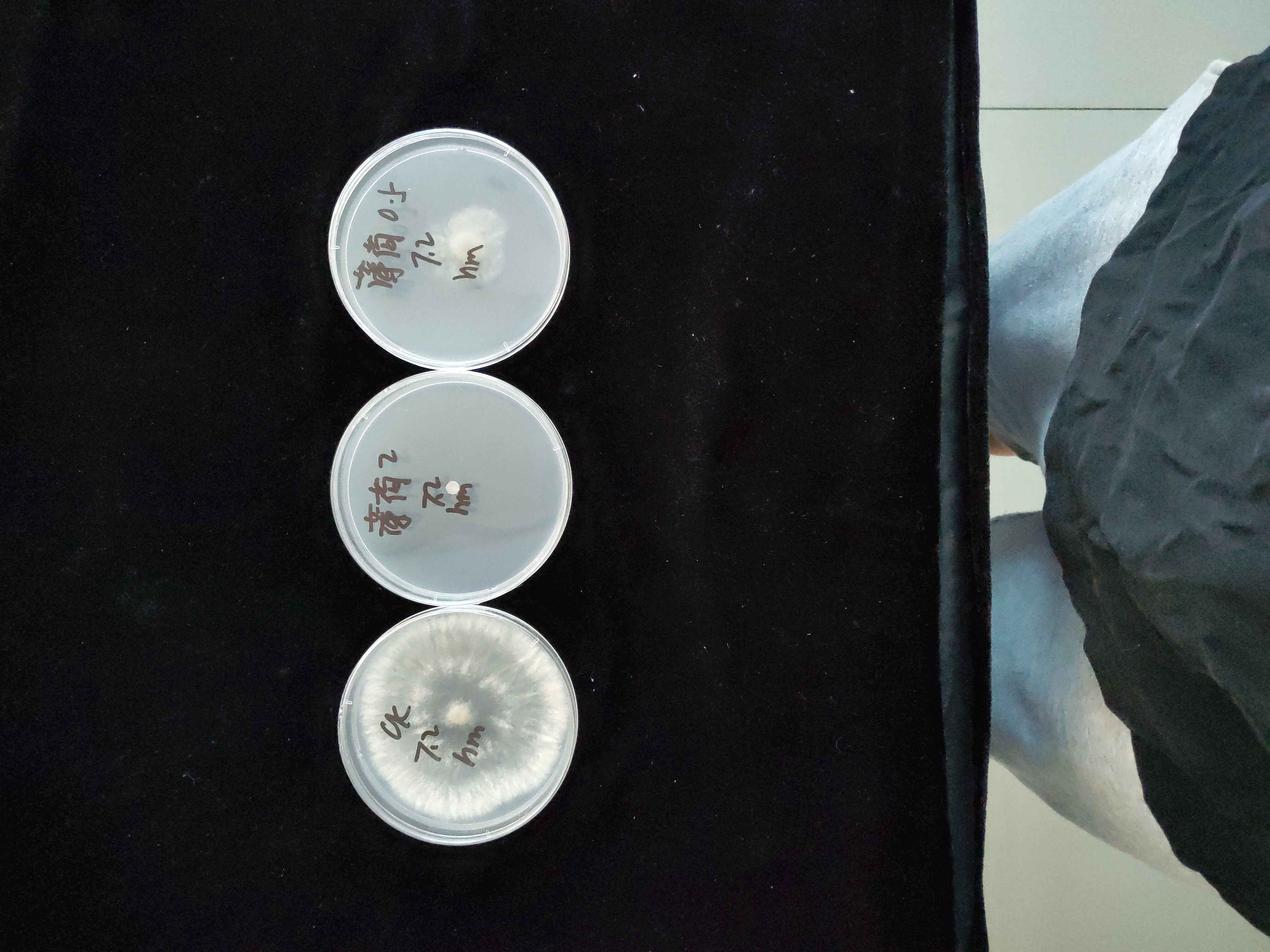

Supplement: Supplemental Information 15 [file peerj-08-9626-s015.zip › Inhibitory activities of 17 plant essential oils on mycelium growth of B. cinerea/photos/Mentha haplocalyx.jpg]

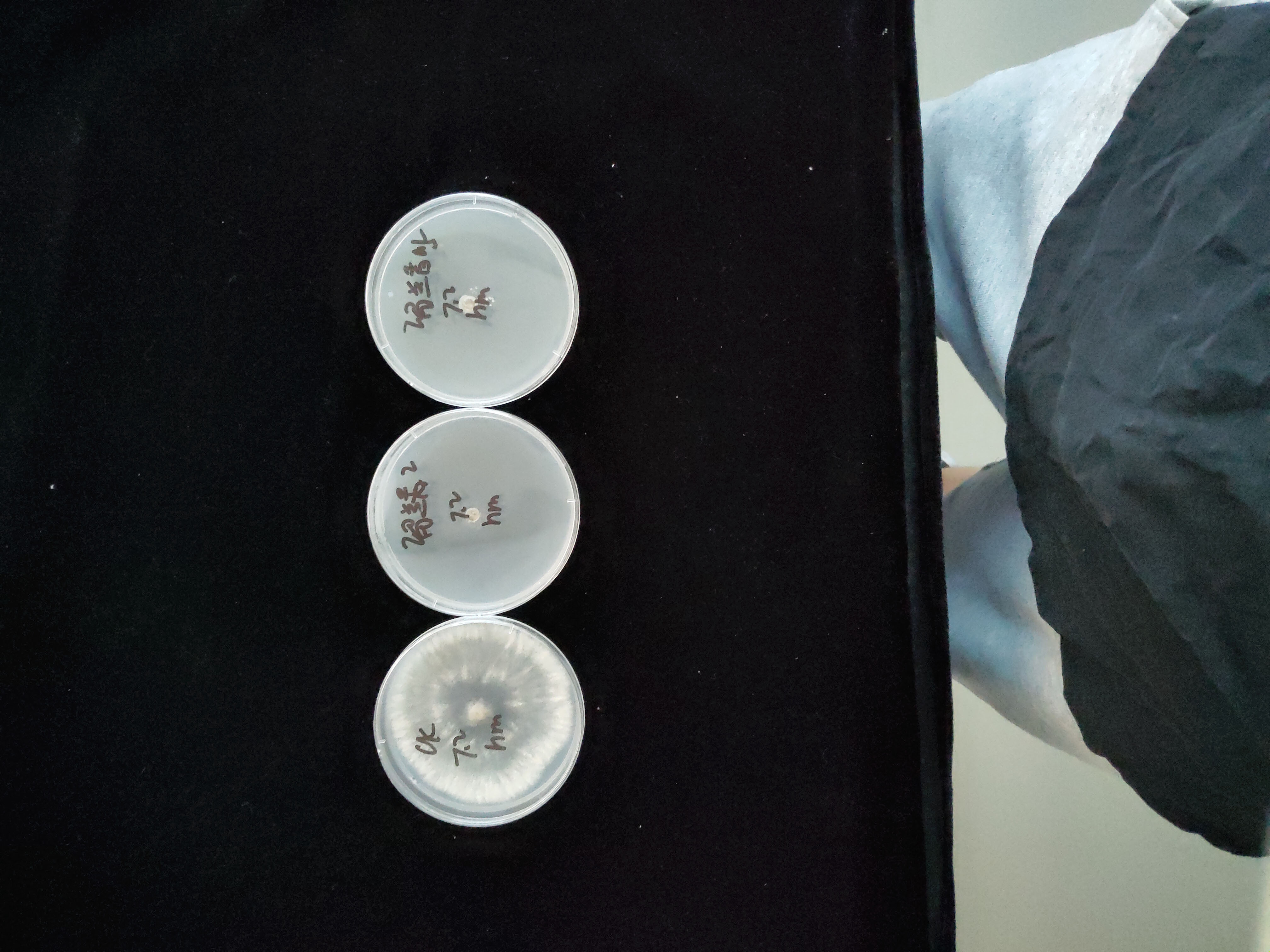

Supplement: Supplemental Information 15 [file peerj-08-9626-s015.zip › Inhibitory activities of 17 plant essential oils on mycelium growth of B. cinerea/photos/Mentha spicata.jpg]

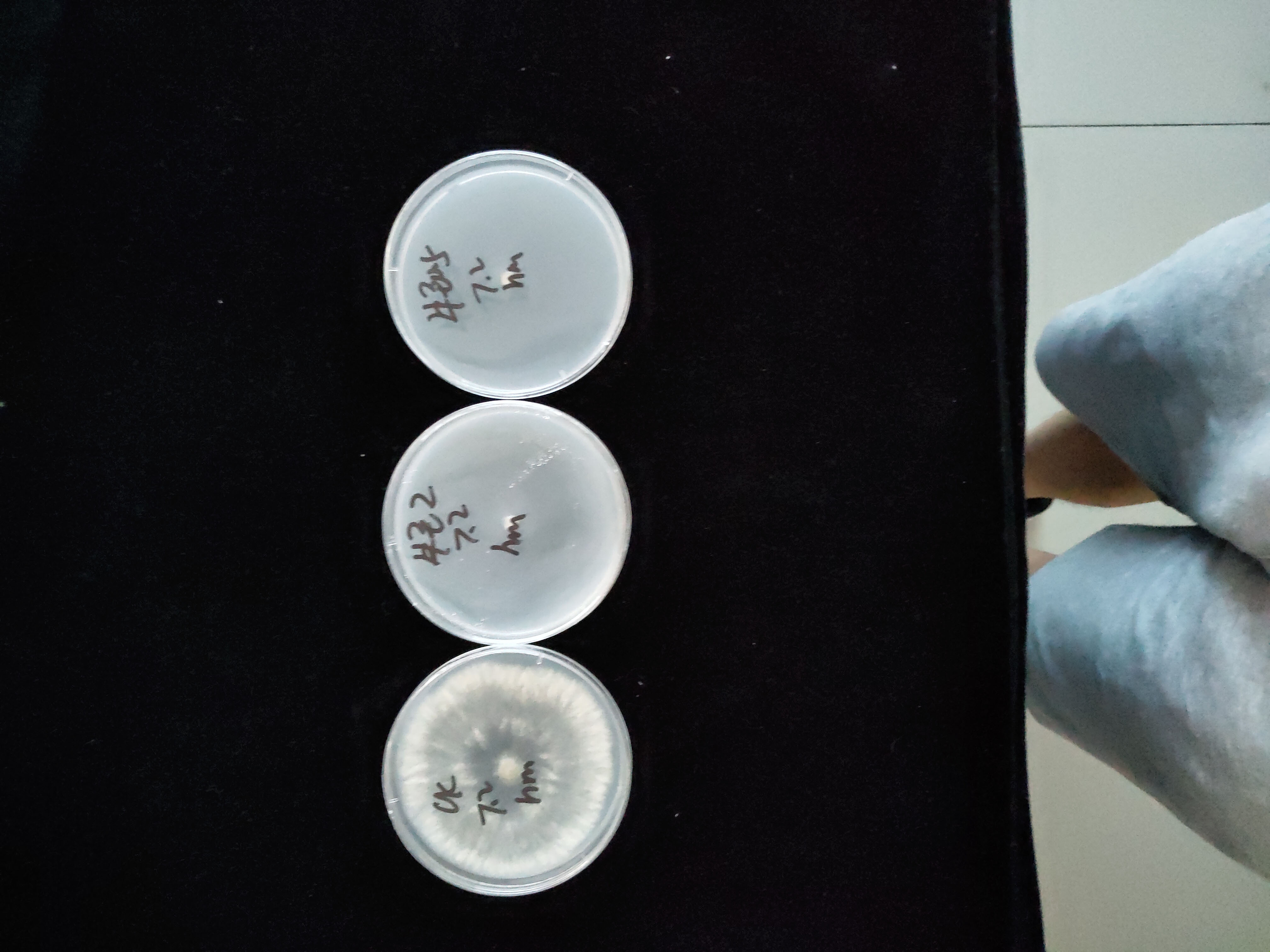

Supplement: Supplemental Information 15 [file peerj-08-9626-s015.zip › Inhibitory activities of 17 plant essential oils on mycelium growth of B. cinerea/photos/Origanum vulgare.jpg]

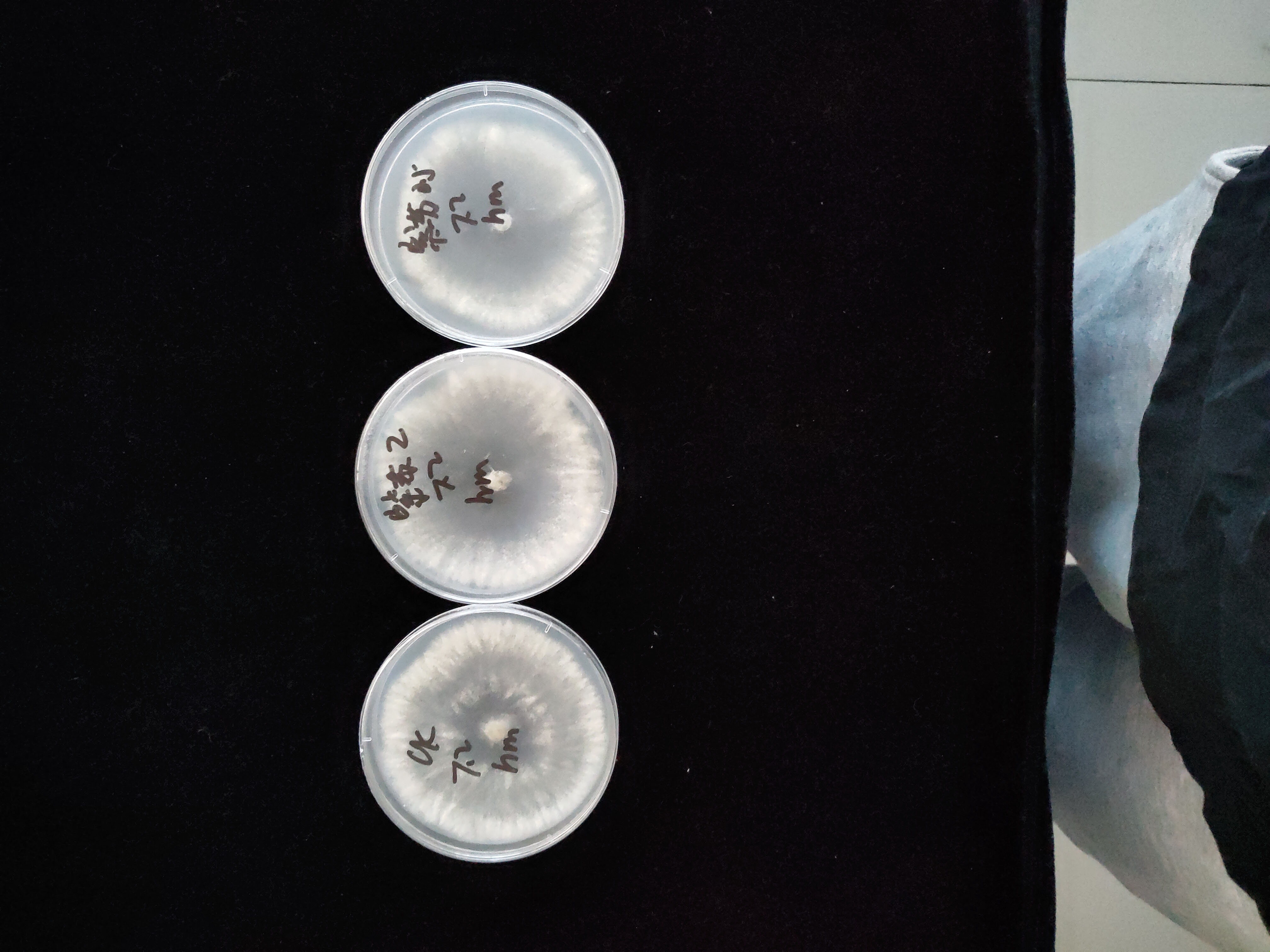

Supplement: Supplemental Information 15 [file peerj-08-9626-s015.zip › Inhibitory activities of 17 plant essential oils on mycelium growth of B. cinerea/photos/Perilla frutescens.jpg]

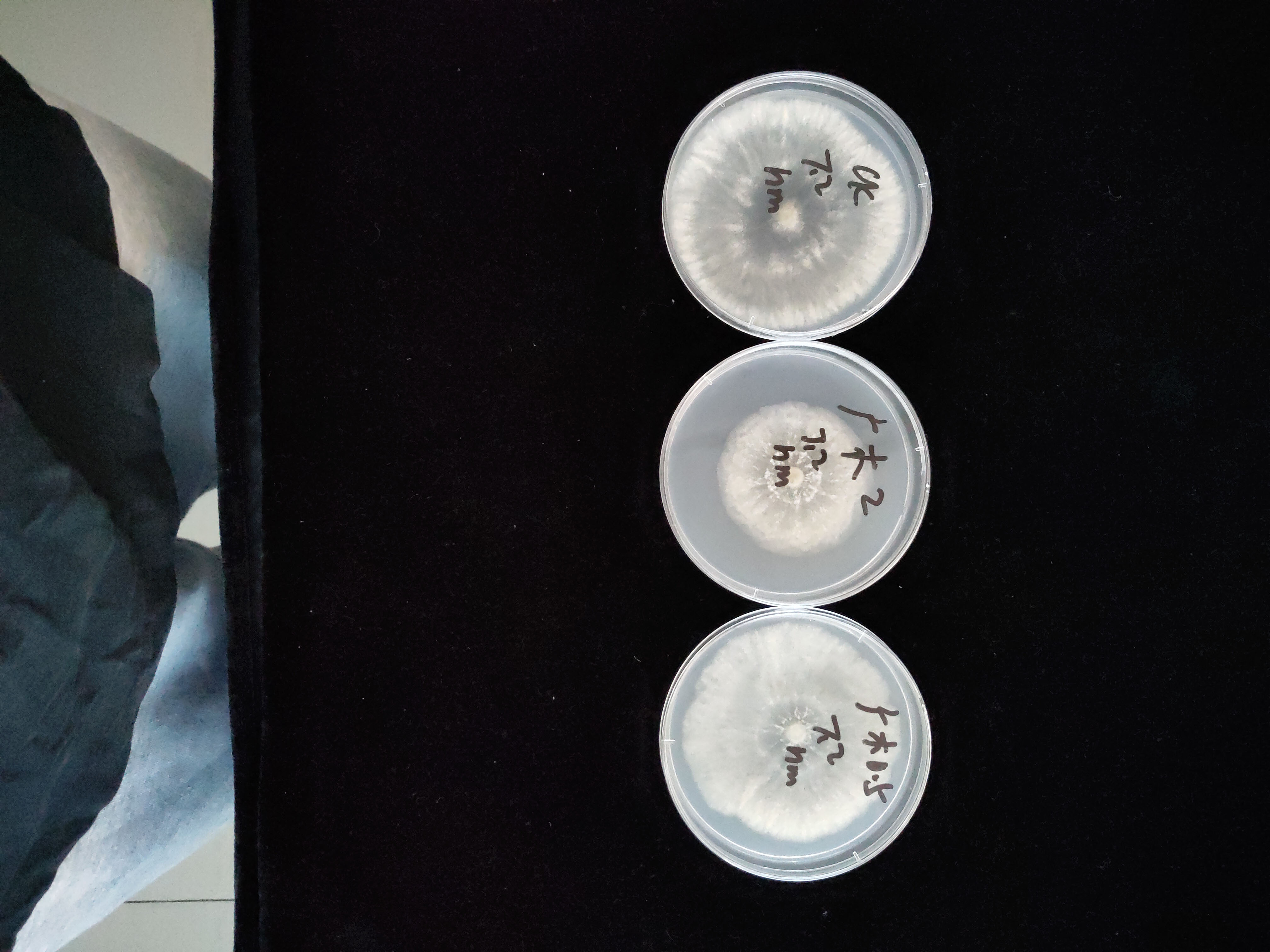

Supplement: Supplemental Information 15 [file peerj-08-9626-s015.zip › Inhibitory activities of 17 plant essential oils on mycelium growth of B. cinerea/photos/Saussurea costus.jpg]

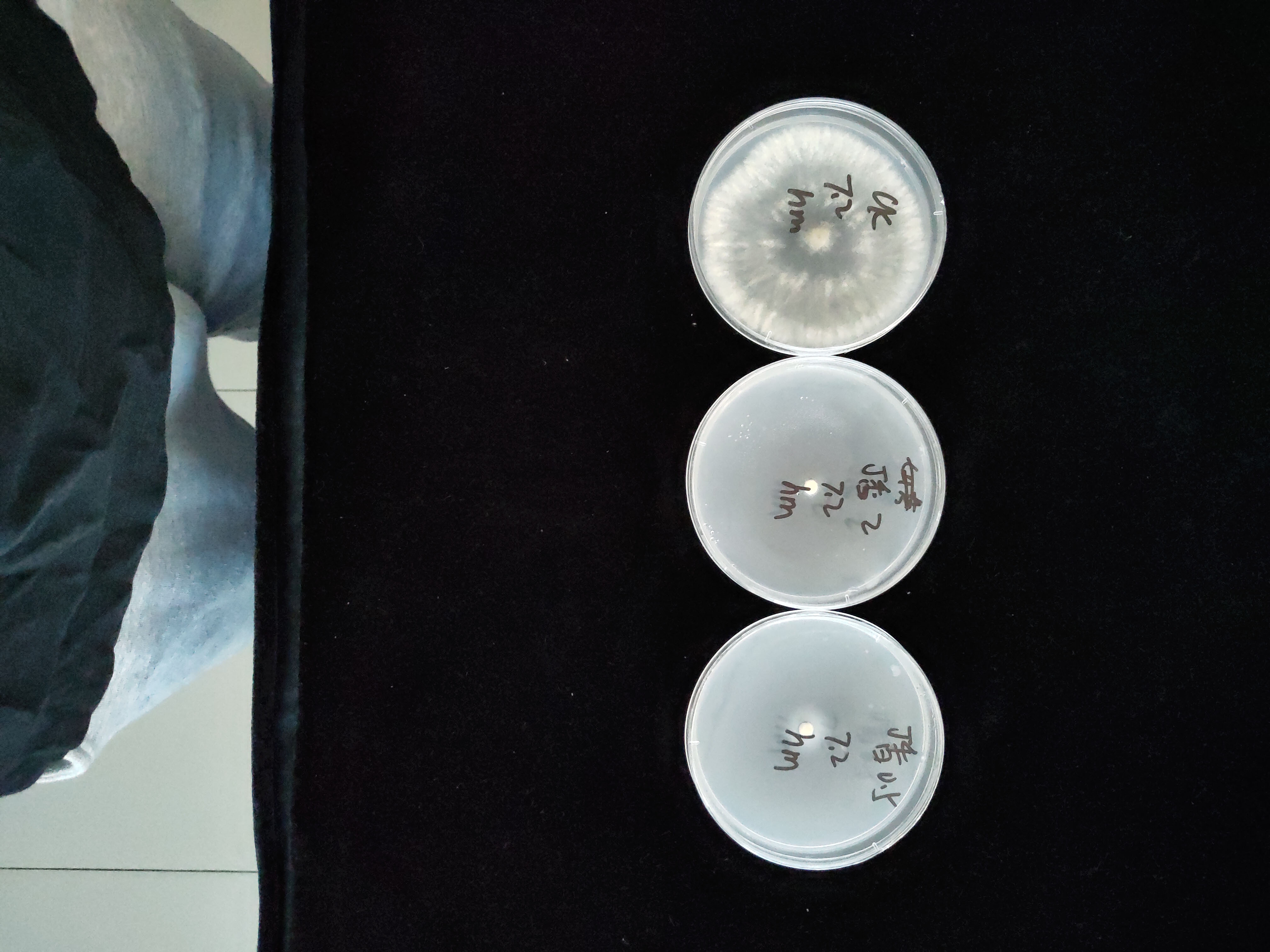

Supplement: Supplemental Information 15 [file peerj-08-9626-s015.zip › Inhibitory activities of 17 plant essential oils on mycelium growth of B. cinerea/photos/Syzygium aromaticum.jpg]

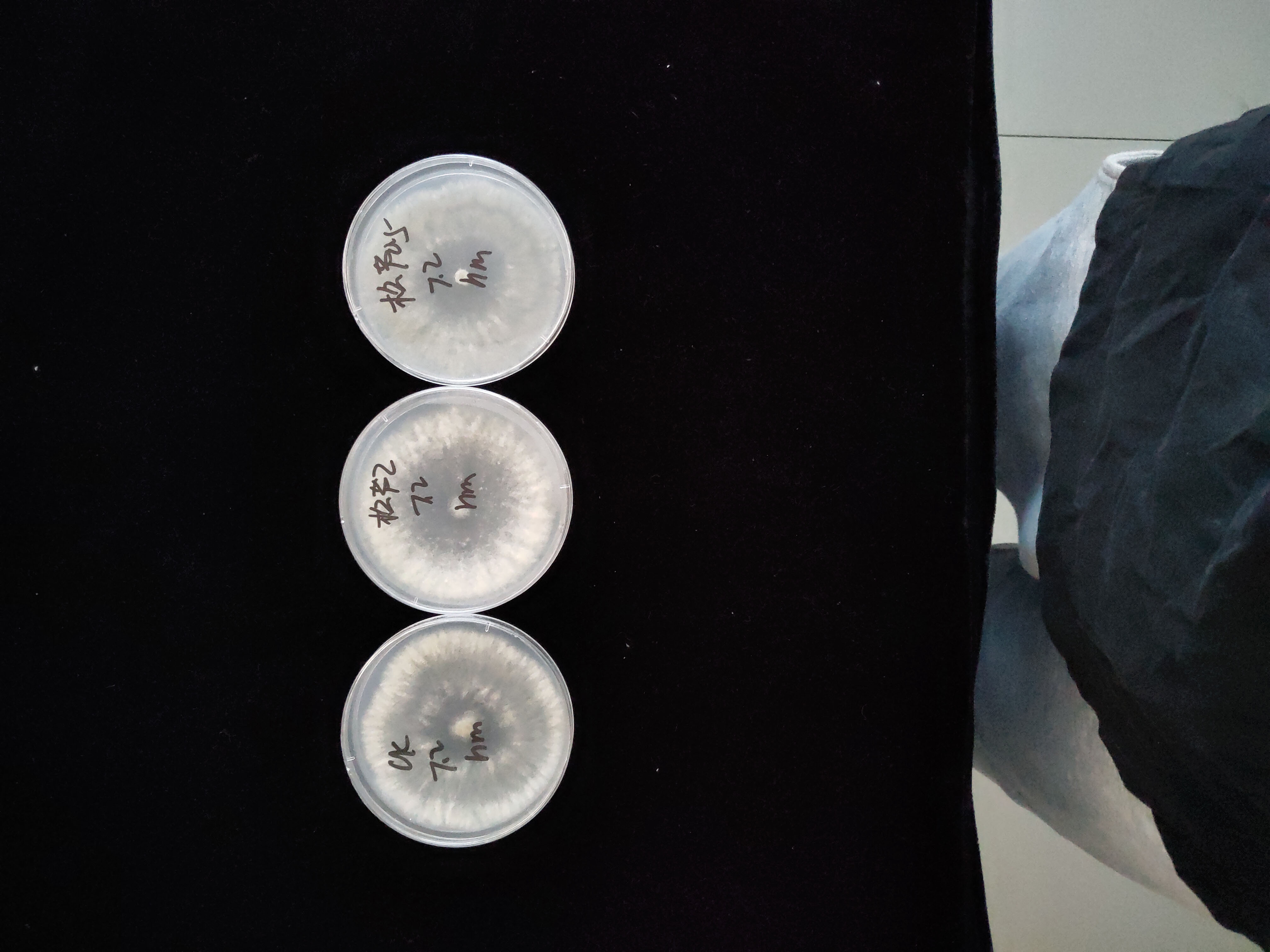

Supplement: Supplemental Information 15 [file peerj-08-9626-s015.zip › Inhibitory activities of 17 plant essential oils on mycelium growth of B. cinerea/photos/turpentine oil.jpg]
