# Supplementary material for: Effects of Origanum vulgare essential oil and its two main components, carvacrol and thymol, on the plant pathogen Botrytis cinerea
Source: PeerJ. 2020 Aug 14;8:e9626. doi: 10.7717/peerj.9626 (PMC7430266; doi:10.7717/peerj.9626)

Qualitative Report

Date File	OVEO 100.D	Date Name	OVEO 100
Date Type		Location	3
Instrument Name	GCMS	User Name	
Collection Method	OVEO 100—Hou Huiyu.M	Acquisition time	2019/4/12 17:27:36(UTC+08:00)
IRM Calibration Status	Not Applicable	Date Method	Qualitative.m
Annotation			
Attenuation	Diluted 100 times	Sample ID	
Sample Name	OVEO 100	Sample Loacation	3
Sample Type		Date File	D:\MassHunter\GCMS\1209Hou Huiyu\ OVEO
Method	D:\DATA\Zhou Lin\ OVEO 100—Hou Huiyu.M	Coverage Method	
Level Name		Annotation	
Balance to Cover		Barcode	
Balance Time (min)	2	Sample Plate Code	
Sample Plate Position		Sample Frame Code	
Sample Frame Position		Expectation Barcode	
Sample Volume		Double injection volume	1
Tuning File	ATUNE.U	Tuning path	D:\MassHunter\GCMS\1\5977\
Tuning Date Time	2019-04-11T12:13:18+08:00	MS Firmware Version	6.00.34
Operator Name		Acquisition Time	2019/4/12 17:27:36 (UTC+08:00)
		Execution completed	True
Sample Locking Operation Mode			
Instrument Name (UTC+08:00)	GCMS	Acquisition Time (Local)	2019/4/12 17:27:36
Acquisition SW Version	MassHunter GC/MS Acquisition B.07.05.2479 23-Aug-2016 Copyright © 1989-2016 Agilent Technologies, Inc.		

Chromatogram

Cracking Voltage	Collision Energy 0	Ion Mode	Unassigned

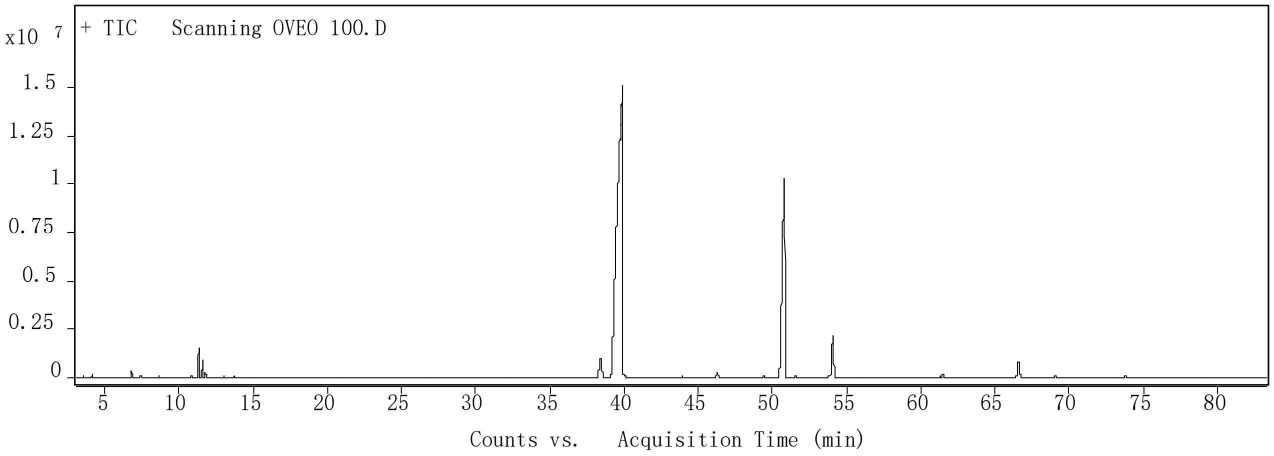

Supplement: Supplemental Information 16 [file peerj-08-9626-s016.pdf]
